# A Phase Ib Study of Sotrastaurin, a PKC Inhibitor, and Alpelisib, a PI3Kα Inhibitor, in Patients with Metastatic Uveal Melanoma

**DOI:** 10.3390/cancers13215504

**Published:** 2021-11-02

**Authors:** Alexander N. Shoushtari, Shaheer Khan, Kimberly Komatsubara, Lynn Feun, Nicolas Acquavella, Shahnaz Singh-Kandah, Tiffany Negri, Alexandra Nesson, Kelly Abbate, Serge Cremers, Elgilda Musi, Grazia Ambrosini, Shing Lee, Gary K. Schwartz, Richard D. Carvajal

**Affiliations:** 1 Memorial Sloan Kettering Cancer Center, New York, NY 10065, USA; abbatek@mskcc.org; 2 Columbia University Irving Medical Center, New York, NY 10032, USA; sk4488@cumc.columbia.edu (S.K.); kmkomatsubara@gmail.com (K.K.); svs2126@cumc.columbia.edu (S.S.-K.); tn2280@cumc.columbia.edu (T.N.); an2843@cumc.columbia.edu (A.N.); sc2752@cumc.columbia.edu (S.C.); em3058@cumc.columbia.edu (E.M.); ga2391@cumc.columbia.edu (G.A.); gks2123@cumc.columbia.edu (G.K.S.); rdc2150@cumc.columbia.edu (R.D.C.); 3 Sylvester Comprehensive Cancer Center, University of Miami, Miami, FL 33136, USA; lfeun@med.miami.edu (L.F.); nacquavella@miami.edu (N.A.); 4 Department of Biostatistics, Mailman School of Public Health, Columbia University, New York, NY 10032, USA; sml2114@cumc.columbia.edu

**Keywords:** uveal melanoma, targeted therapy, sotrastaurin, alpelisib, PKC, PI3K

## Abstract

**Simple Summary:**

Uveal melanoma is a rare subset of melanoma characterized by the presence of early initiating GNAQ/11 mutations, with downstream activation of several pathways which are thought to contribute to cell growth. Based on clinical and preclinical data supporting targeting of protein kinase C (PKC) and the phosphatidylinositol 3-kinase (PI3K) pathway, we conducted a phase Ib study to assess the safety of combined sotrastaurin, a PKC inhibitor, and alpelisib, a PI3K inhibitor. We found that sotrastaurin and alpelisib can be safely administered, however there was no evidence of clinical efficacy.

**Abstract:**

Uveal melanoma (UM) is a rare subset of melanoma characterized by the presence of early initiating GNAQ/11 mutations, with downstream activation of the PKC, MAPK, and PI3Kα pathways. Activity has been observed with the PKC inhibitors sotrastaurin (AEB071) and darovasertib (IDE196) in patients with UM. Inhibition of the PI3K pathway enhances PKC inhibition in in vivo models. We therefore conducted a phase Ib study of sotrastaurin in combination with the PI3Kα inhibitor alpelisib to identify a tolerable regimen that may enhance the activity of PKC inhibition alone. Patients with metastatic uveal melanoma (n = 24) or GNAQ/11 mutant cutaneous melanoma (n = 1) were enrolled on escalating dose levels of sotrastaurin (100–400 mg BID) and alpelisib (200–350 mg QD). The primary objective was to identify the maximum tolerated dose (MTD) of these agents when administered in combination. Treatment-related adverse events (AE) occurred in 86% (any grade) and 29% (Grade 3). No Grade 4–5-related AEs occurred. Dose Level 4 (sotrastaurin 200 mg BID and alpelisib 350 mg QD) was identified as the maximum tolerated dose. Pharmacokinetic analysis demonstrated increasing concentration levels with increasing doses of sotrastaurin and alpelisib, without evidence of interaction between agents. Pharmacodynamic assessment of pMARCKS and pAKT protein expression with drug exposure suggested modest target inhibition that did not correlate with clinical response. No objective responses were observed, and median progression-free survival was 8 weeks (range, 3–51 weeks). Although a tolerable dose of sotrastaurin and alpelisib was identified with pharmacodynamic evidence of target inhibition and without evidence of a corresponding immunosuppressive effect, limited clinical activity was observed.

## 1. Introduction

Uveal melanoma is a rare subtype of melanoma that accounts for approximately 3–5% of all melanomas [1,2]. Treatment approaches that have demonstrated efficacy in advanced cutaneous melanoma have been less effective in uveal melanoma due to its distinct biology and clinical behavior. Although the recently reported data of tebentafusp, a bifunctional fusion protein targeting gp100 in an HLA-restricted fashion, as well as CD3, in HLA-A0201 positive patients with metastatic uveal melanoma demonstrates that immune-based treatment approaches meaningfully impact overall survival, to date, no molecularly targeted approach has been found to do the same in a randomized controlled setting [3,4].

Uveal melanomas generally harbor early initiating mutations in genes encoding for the G-α-protein-subunits, *GNAQ* or *GNA11*, with the remainder of cases harboring other alterations, such as mutations in CYSLTR2 or PLCB4, which functionally activate the G-α pathway [5,6]. Mutations in *GNAQ* and *GNA11* result in disabling of their intrinsic GTPase activity, leading to cleavage of phosphatidylinositol diphosphate (PIP_2_) into inositol triphosphate (IP_3_) and DAG, and subsequent activation of downstream effectors including protein kinase C (PKC), a mediator of the mitogen-activated protein kinase (MAPK) pathway [7,8]. The PKC family is a group of serine/threonine kinases composed of different isoforms that are subdivided into classical, novel, and atypical isoforms, each with different functions [9]. PKC isoforms are involved in regulation of oncogenesis via activation of various pathways, including the MAPK/ERK1/2 pathway. A number of preclinical studies have demonstrated significant anti-tumor effects of inhibition of various PKC isoforms in uveal melanoma models [10,11].

In a completed phase I study of sotrastaurin, a potent, selective oral inhibitor of both the classical and novel isoforms of PKC, 153 patients with metastatic uveal melanoma received total daily doses of sotrastaurin ranging from 450 to 1400 mg on a twice daily or three times daily dosing schedule [12]. The recommended monotherapy dose of sotrastaurin was 1400 mg per day for a BID dosing schedule (700 mg BID) and 750 mg/day for a TID dosing schedule (250 mg TID). Although target inhibition was demonstrated in the majority of patients following 2 weeks of dosing, clinical activity was modest, with a 3% overall response rate by RECIST criteria and a median progression-free survival of 3.5 months.

We previously demonstrated the induction of phospho-AKT with exposure to sotrastaurin in preclinical uveal melanoma models, suggesting upregulation of the PI3K/AKT pathway as a mechanism of resistance to PKC inhibition [13]. Combined exposure of sotrastaurin with alpelisib, a selective PI3K-alpha inhibitor, resulted in synergistic cell death in *GNAQ* and *GNA11* mutant uveal melanoma cell lines and tumor growth inhibition in a *GNAQ*-mutant xenograft tumor model. Furthermore, suppression of both the PKC/ERK and PI3K/AKT pathways was observed with combined treatment in vitro and in vivo [13]. Based on this preclinical data, we hypothesized that concurrent PKC and PI3K inhibition would lead to improved clinical outcomes when compared with PKC inhibition alone, and initiated this phase Ib, multi-center, open-label clinical trial to evaluate the clinical safety and tolerability, preliminary efficacy, pharmacokinetics, and pharmacodynamics of the combination of sotrastaurin and alpelisib in patients with advanced uveal melanoma.

## 2. Materials and Methods

### 2.1. Clinical Study Design and Treatment

This was a phase Ib, open-label, multicenter, single-arm study conducted at Columbia University Irving Medical Center (New York, NY, USA), Memorial Sloan Kettering Cancer Center (New York, NY, USA), and the University of Miami (Miami, FL, USA). This study was conducted in accordance with the Declaration of Helsinki ethical principles, Good Clinical Practices, principles of informed consent, and requirements for public registration of clinical trials (ClinicalTrials.gov identifier, NCT02273219). Site-specific Institutional Review Boards approved the protocol and written informed consent was obtained from each subject at enrollment.

The primary objective of this study was to determine the maximum tolerated dose (MTD) for the combination of sotrastaurin and alpelisib in patients with metastatic uveal melanoma or other melanoma subtypes harboring a mutation in GNAQ or GNA11. Secondary objectives were to evaluate the efficacy of combined treatment with sotrastaurin and alpelisib as determined by investigator-assessed objective response rate (ORR) using RECIST v1.1 criteria, investigator-assessed progression-free survival (PFS), and overall survival (OS), and to evaluate the pharmacokinetic properties of combined treatment with sotrastaurin and alpelisib at varying dose levels. Exploratory objectives included pharmacodynamic analysis assessing target inhibition of the PKC and PI3K pathway via Western blot analysis and gene-expression profiling on paired tumor biopsies to assess changes in tumor gene expression. All the whole western blot figures can be found in the supplementary materials.

Subjects received sotrastaurin (100–400 mg twice a day) and alpelisib (200–350 mg daily) administered orally on a continuous 28-day cycle (Appendix A Table A1). Within each cohort, a 14-day observation period was required after enrollment of the first subject and before the next subject could be enrolled in the cohort. Between cohorts, all subjects were required to complete the 28-day cycle before opening the next cohort, provided the prior dose level was determined to be tolerable. A 3 + 3 study design was utilized to estimate the MTD, with optional expansions of a particular dose level using a 3 + 3 + 3 approach to further investigate safety and toxicity at sponsor–investigator discretion. If 0 out of 3 or 0–1 out of 6 patients (or 0–2 out of 9 patients in the case of a 3 + 3 + 3 expansion) experienced a dose-limiting toxicity (DLT) as defined by the protocol, the dose level was escalated. If 2 or more DLTs were observed (or 3 or more DLTs in the case of a 3 + 3 + 3 expansion), the highest tolerated dose was defined as 1 dose level below that level.

### 2.2. Patient Selection

Eligible patients were required to have a histologically confirmed diagnosis of metastatic uveal melanoma or other melanoma subtype harboring a mutation in GNAQ or GNA11. There were no restrictions on number or type of prior therapies. Eligible patients were ≥18 years old, had an ECOG performance status of 0–2, and had measurable disease by RECIST v1.1 criteria. Due to the known association of alpelisib with hyperglycemia, patients with a history of Type I Diabetes Mellitus (DM) or Type II DM requiring insulin were excluded, and fasting plasma glucose had to be <140 mg/dL in all patients. The study was later amended to exclude all patients with a history of Type II DM.

### 2.3. DLT Definition

Toxicity was graded using NCI Common Terminology Criteria for Adverse Events (CTCAE v. 4.0). For recurrent adverse events, the highest reported grade per event per patient was assessed. DLTs were defined as any treatment-related Grade 3 or higher toxicity, or intolerable Grade 2 toxicity that required a greater than 21-day treatment delay, occurring during the first 28 days of study therapy. In addition, specific DLT criteria were defined for the following adverse events: Grade 3 neutropenia and fever, Grade 3 anemia only if due to a hemolytic process related to study treatment, Grade 3 thrombocytopenia and bleeding, for subjects with liver metastases, Grade 3 AST or ALT only if ≥25% rise from baseline and not definitely due to disease progression, and Grade 3 diarrhea ≥ 48 h despite maximum prophylaxis. Patients who did not complete the 28-day DLT evaluation period were considered unevaluable for the purpose of DLT evaluation and efficacy.

### 2.4. Evaluation of Clinical Activity

Imaging was obtained at baseline, at 4 and 8 weeks after the start of study treatment, and then every 8 weeks thereafter. A CT of the chest, and CT or MRI of the abdomen and pelvis with contrast were required at each time point, with additional modalities of imaging permitted at investigator discretion. Reponses were assessed using RECIST v.1.1 criteria by investigator assessment. Criteria for removal from study included radiographic or clinical disease progression, or unacceptable toxicity.

### 2.5. Correlative Studies

Pharmacokinetic (PK) Analysis: Plasma samples were collected for PK analysis during Cycle 1 on Day 1 (pre-dose, 0.5, 1, 2, 4, 6, 8, 24 h post-dose), Day 8 (pre-dose, 0.5, 1, 2, 4, 6, 8 h post-dose), Day 15 (pre-dose), and pre-dose on Day 1 of Cycles 2–6. Plasma concentration levels of sotrastaurin and its metabolite AEE800 were assessed in all subjects. Plasma concentration levels of alpelisib were performed in a cohort of patients who were enrolled at Columbia University Irving Medical Center. PK analysis was performed using a previously validated LC-MS/MS procedure (WuXi AppTec, Shanghai, China). The reference standards for sotrastaurin, AEE800, and alpelisib were supplied by Novartis.

Pharmacodynamic (PD) Analysis: The protocol was amended to mandate paired tumor biopsies for PD analysis in all enrolled subjects starting in April 2016 (during enrollment of Dose Level 4). Paired tumor biopsies with sufficient flash-frozen tissue for analysis were subsequently obtained at baseline (day −28 to −1) and at Cycle 1 Day 15 (±3 days) in a total of 7 patients. Tumor biopsies were lysed in radioimmunoprecipitation assay (RIPA) buffer supplemented with a protease inhibitor cocktail tablet (Roche Diagnostics) and 1 mmol/L of Na_3_VO_4_. Equal amounts of protein were loaded and separated on a 4–12% PAGE gel (Invitrogen). Proteins were transferred to polyvinylidenedifluoride (PVDF) membranes, which were blocked in 5% nonfat dried milk. Membranes were then incubated with primary and secondary antibodies and developed by ECL. Antibodies used to probe were: pAKT (Ser473, #4060, Clone D9E), Pan AKT (#2920, Clone 40D4), pS6 (S240/244, #4858, Clone D57.2.2E), S6 ribosomal protein (Ser235/236, #2317, Clone 54D2), pMARCKS (Ser152/156, #2741), MARCKS (#5607, Clone D88D11), GAPDH (#5174, Clone D16HI1), pERK1/2 (Y204, #4370, Clone), ERK1/2 (#4695, Clone 137F5), Cyclin D1(#2978, Clone 92G2), Cyclin E1 (#20808, Clone D7T3U), Cyclin A2 (#67955, Clone E6D1J), Bcl-2 (#4223, Clone D55G8), and GLUT4 (Santa Cruz Biotech #SC-53566, Clone IF8), obtained from Cell Signaling Technology Inc., unless otherwise noted.

Gene Expression Analysis: RNA was extracted from flash-frozen paired tumor biopsy samples with the RNeasy Micro Kit (Qiagen) according to the manufacturer’s protocol and quantified using the NanoDrop spectrophotometer. Gene expression analysis was performed using the PanCancer IO360^TM^ panel (Nanostring Technologies) to assess 770 immune-related genes in 7 paired tumor biopsy samples. A total of 50 ng of RNA per sample was mixed with a 3′ biotinylated capture probe and a 5′ reporter probe tagged with a fluorescent barcode from the custom gene expression code set. Probes and target transcripts were hybridized at 65 °C for 16 h and then run on the NanoString nCounter^TM^ platform according to the manufacturer’s protocol. The samples were scanned at maximum scan resolution on the nCounter Digital Analyzer and data were analyzed using nSolver Analysis Software. Gene expression counts were normalized using the geometric mean of housekeeping genes included in the panel selected by the GeNorm algorithm. Differential gene expression in the on-treatment samples was compared with baseline gene expression in pre-treatment samples. Pathway scores were analyzed using the nSolver Analysis Software, which is based on previously described methods [14].

## 3. Results

### 3.1. Patient Demographics

Between November 2014 and November 2017, a total of 25 patients were enrolled, and 24 patients were evaluable for DLT and efficacy. One patient in dose level 4 did not complete the DLT observation period due to rapid disease progression (Day 14), was deemed unevaluable for DLT and efficacy, and was replaced. Twenty-four patients had a diagnosis of uveal melanoma and one patient had melanoma of unknown primary harboring a GNAQ mutation (Table 1). The median age of enrolled patients was 62 years old (range, 25–78) and the median number of prior systemic therapies was 3 (range, 0–8). The majority of patients harbored a GNAQ or GNA11 mutation (14 (56%) GNAQ mutant; 8 (32%) GNA11 mutant). Three patients (12%) were wildtype for GNAQ and GNA11. Mutational status for PLCB4, CYSLTR2, BAP1, SF3B1, or EIFA1X was not available.

### 3.2. Dose Escalation and MTD Determination

Of the 3 initial patients enrolled in dose level 1 (sotrastaurin 100 mg BID and alpelisib 200 mg daily), 1 patient experienced Grade 3 hyperglycemia attributed to study therapy and was declared a DLT. An additional 3 patients were enrolled into dose level 1 without further DLTs observed, however 1 patient experienced Grade 3 nausea and vomiting soon after the end of the DLT period (during Cycle 2 Week 1). An additional 3 patients were subsequently enrolled into dose level 1 (total of 9 patients) for additional safety information. One patient in this group experienced Grade 3 nausea related to study therapy meeting DLT criteria, resulting in 2 patients out of 9 experiencing a DLT in dose level 1 (Table 2). No DLTs occurred in dose level 2 (sotrastaurin 200 mg BID and alpelisib 250 mg daily) or dose level 3 (sotrastaurin 200 mg BID and alpelisib 300 mg daily). In dose level 4 (sotrastaurin 200 mg BID and alpelisib 350 mg daily), 3 patients were initially enrolled with no DLTs observed, and the dose level was subsequently escalated to dose level 5 (sotrastaurin 300 mg BID and alpelisib 350 mg daily). In dose level 5, 2 of the 3 patients enrolled experienced DLTs (intolerable Grade 2 fatigue, diarrhea and nausea (n = 1), and Grade 3 neutropenia > 7 days (n = 1)), and dose level 5 was declared intolerable. Subsequently, an additional 3 patients were enrolled into dose level 4 to obtain additional safety information. One patient experienced intolerable Grade 2 fatigue requiring a dose reduction, for a total of 1 patient out of 6 experiencing a DLT in dose level 4. Dose level 4 (sotrastaurin 200 mg BID and alpelisib 350 mg daily) was declared the maximum tolerated dose.

### 3.3. Clinical Safety

Treatment-related adverse events (AE) occurred in 86% (any grade) and 29% (Grade 3) of patients (Table 3). No Grade 4–5 treatment-related AEs occurred. The most frequent observed AEs were GI-related, including diarrhea (60% (any grade) and 8% (Grade 3)) and nausea (64% (any grade), 8% (Grade 3)). Fatigue occurred in 52% of patients (52% (any grade), 8% (Grade 3)) and anorexia in 40% (40% (any grade), 4% (Grade 3)). Hyperglycemia occurred in 40% of patients (40% (any grade), 8% (Grade 3)). One of the patients with Grade 3 hyperglycemia had a known history of Type II DM that was previously controlled on oral medications. Rash occurred in 20% of patients (16% (grade 1), 4% (grade 2)).

A total of 8 patients (32%) required dose reductions for treatment-related toxicity. Dose reductions due to AEs occurred in 4 out of the 5 patients who experienced DLTs, and 1 patient with a DLT discontinued treatment due to the AE. Notably, 4 patients required dose reductions for late toxicities that occurred after the end of the 28-day DLT observation period. Late dose reductions occurred in 1 patient each in dose level 2 (Grade 2 intolerable fatigue), dose level 3 (Grade 2 intolerable fatigue and nausea), dose level 4 (Grade 2 intolerable nausea and anorexia), and dose level 5 (Grade 2 intolerable fatigue).

### 3.4. Clinical Efficacy

**Figure 1:**
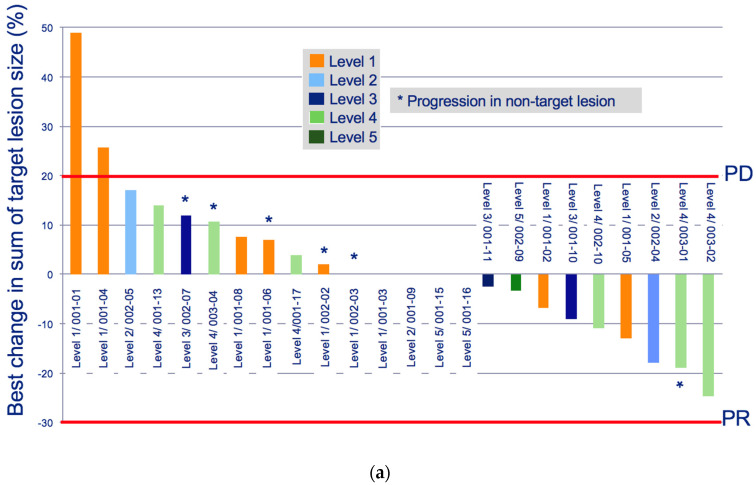

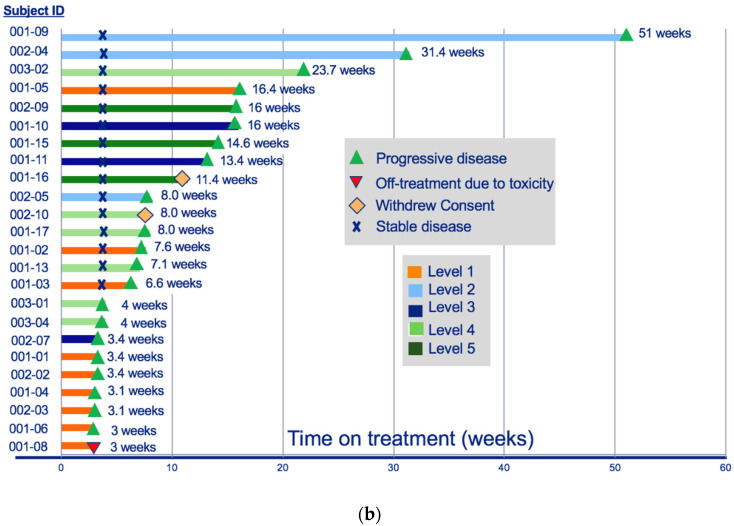
Clinical response to treatment: (**a**) best % change in sum of target lesions by investigator-assessed RECIST response, and (**b**) duration of treatment.

**Figure A1:**
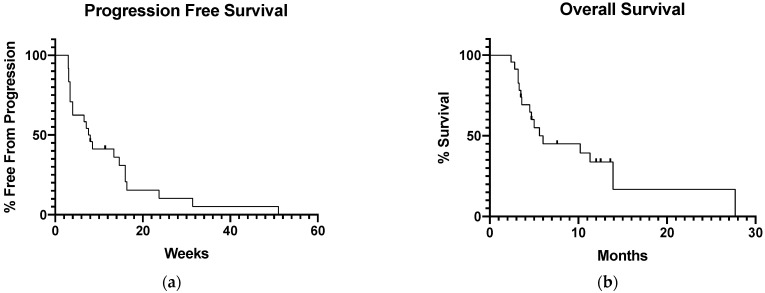
Kaplan-Meier estimates of progression-free survival (**a**) and overall survival (**b**) in all evaluable patients. The vertical lines indicate that patients’ data were censored. The median progression-free survival was 7.6 weeks (range, 3–51 weeks).The median overall survival was 6.0 months (range, 2.4–27.7 months).

A total of 24 patients were evaluable for response. No complete or partial responses by RECIST v1.1 criteria were observed. A best response of stable disease was observed in 66.6% (n = 16) of patients at 4 weeks from treatment initiation and 37.5% (n = 9) at 8 weeks. The median investigator-assessed progression-free survival was 7.6 weeks (range, 3–51 weeks) (Table 4, Appendix A Figure A1). The median overall survival was 6.0 months (range, 2.4–27.7 months) (Appendix A Figure A1). Stable disease was observed as the best response across all dose levels, without a clear dose–response association (Figure 1a,b). One patient in dose level 2 achieved durable stable disease lasting for 51 weeks and two additional patients in dose level 2 and dose level 4 respectively achieved stable disease lasting longer than 20 weeks. Baseline characteristics for these three patients are described in Appendix A Table A2.

### 3.5. Correlative Analysis

#### 3.5.1. Pharmacokinetic Analysis: Sotrastaurin, AEE800, and Alpelisib Concentration over Time

**Figure 2:**
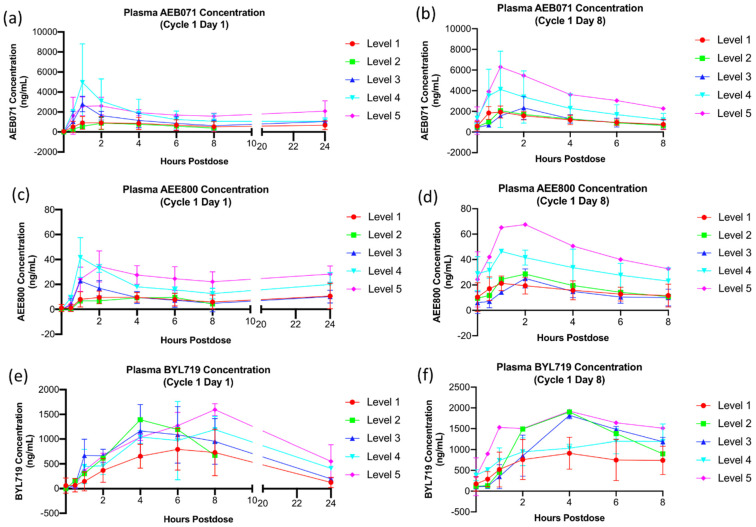
Plasma concentrations of sotrastaurin, AEE800, and alpelisib over time. Mean plasma concentration at Cycle 1 Day 1 (0, 0.5, 1, 2, 4, 6, 8, 24 h post-dose) and Cycle 1 Day 8 (0, 0.5, 1, 2, 34, 6, 8 h post-dose) is graphed by dose level. Error bars represent standard deviation (SD). (**a**) Sotrastaurin concentration (ng/mL) on Cycle 1 Day 1. (**b**) Sotrastaurin concentration (ng/mL) on Cycle 1 Day 8, (**c**) AEE800 (ng/mL) concentration on Cycle 1 Day 1. (**d**) AEE900 (ng/mL) concentration on Cycle 1 Day 8. (**e**) Alpelisib (ng/mL) concentration on Cycle 1 Day 1. (**f**) Alpelisib (ng/mL) concentration on Cycle 1 Day 8.

Evaluation of plasma sotrastaurin concentration over time, although limited by small and varying sample sizes across doses, demonstrated increasing sotrastaurin plasma concentrations with higher doses (Figure 2a,b). Plasma concentration of AEE800 (Figure 2c,d), the primary metabolite of sotrastaurin, demonstrated a similar pattern, with increasing concentration observed with higher sotrastaurin dose levels. Maximum concentration of sotrastaurin was reached rapidly within 2 h after dosing. The average half-life of sotrastaurin was 5.7 h on day 1 and 5.1 h on day 7. Accumulation of systemic exposure was modest, with an average accumulation ratio of 1.6 from day 1 to day 7. There was little substantial further accumulation in systemic exposure, as evidenced by the apparent lack of increase in the pre-dose concentration of sotrastaurin and AEE800 observed over time (Figure 2g). Pharmacokinetic analyses on alpelisib samples were performed on a limited cohort of patients (n = 13). Plasma concentration of alpelisib (Figure 2e,f) demonstrated increasing drug plasma levels with increasing dose levels. The maximum concentration of alpelisib was reached over a more extended time after dosing compared to sotrastaurin, approaching 6 h. The average half-life of alpelisib was 7.6 h on day 1 and 7.8 h on day 7. Accumulation of systemic exposure was modest, with an average accumulation ratio of 1.3 from day 1 to day 7. There was some accumulation in the pre-dose concentration of alpelisib observed over time, with maximum concentrations seemingly reached at the third cycle (Table 5). Pharmacokinetic parameters of both drugs are listed in detail in Appendix A Table A3.

Plasma concentration levels of sotrastaurin in this study demonstrated a similar pattern to previously published levels seen with sotrastaurin monotherapy, suggesting a limited interaction between sotrastaurin and alpelisib [15].

#### 3.5.2. Pharmacodynamic Analysis: PKC and PI3Kα-AKT-mTOR Pathway Inhibition

**Figure 3:**
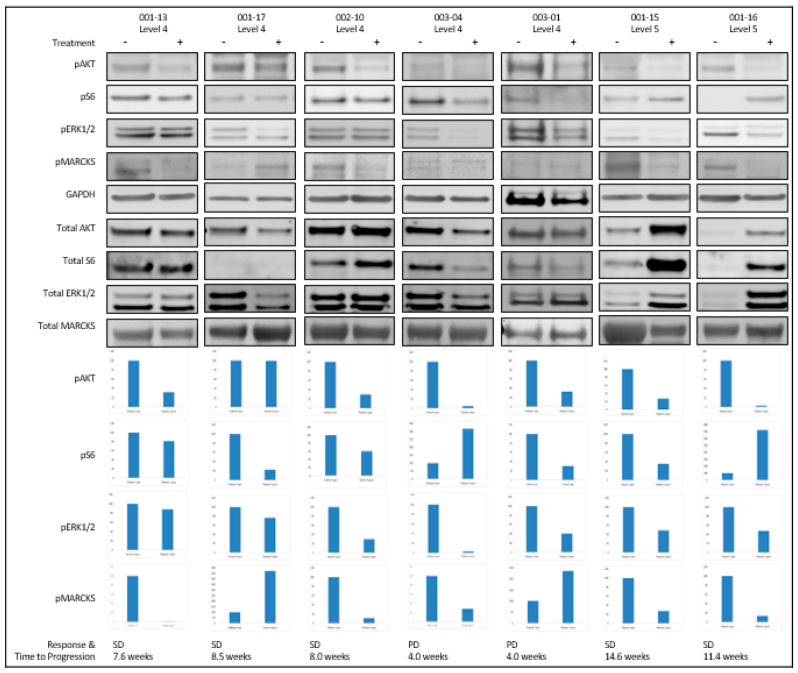
Western blot analysis of PKC and PI3Kα-AKT-mTOR pathway inhibition. Paired tumor biopsies were assessed by Western blot for pAKT, pS6, pERK1/2, pMARCKS, and the respective total proteins. GAPDH was used as a loading control. Clinical outcomes for these respective patients are listed. Protein quantitation of the pre-treatment (left) and post-treatment (right) Western blot analysis performed with Image J. The pre-treatment sample expression level represents a baseline of 100%, with the post-treatment sample expression levels relative to this baseline. Bar plots represent pAKT/total AKT, pS6/total S6, pERK1/2/ total ERK1/2, and pMARCKS/MARCKS, respectively.

To evaluate target inhibition of PKC with sotrastaurin, MARCKS and pMARCKS protein levels were evaluated by Western blot analysis on available paired tumor biopsy specimens. MARCKS is a known substrate of PKC, and preclinical studies have demonstrated that inhibition of PKC results in reduced pMARCKS levels [16]. A decrease in pMARCKS/MARCKS levels was observed upon treatment in both patients in dose level 5, and 4 out of 5 patients in dose level 4. Additionally, pERK1/2/ERK1/2 levels were decreased upon treatment in all patients, consistent with previous reports demonstrating that sotrastaurin inhibits ERK phosphorylation in GNAQ-mutant cells lines [11]. To evaluate target inhibition of the PI3Kα-AKT-mTOR pathway with alpelisib, pAKT and pS6 and corresponding total protein levels were evaluated by Western blot analysis on the same paired tumor biopsy specimens. Inhibition of pAKT/AKT was observed upon treatment in both patients in dose level 5, and 4 out of 5 patients in dose level 4. pS6/S6 levels were suppressed in 4 out of 5 patients on dose level 4 and one patient treated on dose level 5 (Figure 3).

Despite suppression of pMARCKS, pERK1/2, pAKT, and pS6, none of the patients with paired biopsies (n = 7) derived clinical benefit. Clinical characteristics and outcomes for these corresponding 7 patients are detailed in Appendix A Table A4.

**Figure A2:**
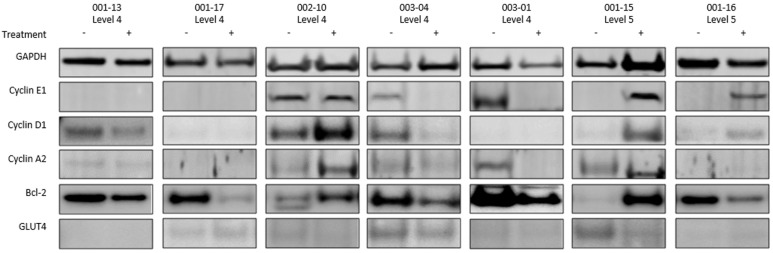
Supplemental Western blot analyses for potential markers of cell proliferation, apoptosis, and metabolic activity. Paired tumor biopsies were assessed by western blot for Cyclin E1, D1, A2, Bcl-2, and GLUT4. GAPDH was used as a loading control.

To further assess the treatment effect on proteins associated with proliferation, apoptosis, and metabolic activity, cyclin levels (D1, E1, A2), Bcl-2, and GLUT-4 were also evaluated by Western blot. Although Bcl-2, an anti-apoptotic protein known to be upregulated in uveal melanoma, was decreased in 5 of 7 samples, no clear pattern was seen with markers of proliferation or metabolic activity to further explain the lack of tumor response with treatment. Results are shown in Appendix A Figure A2.

#### 3.5.3. Effect of Sotrastaurin and Alpelisib on Immune-Related Gene Expression

**Figure 4:**
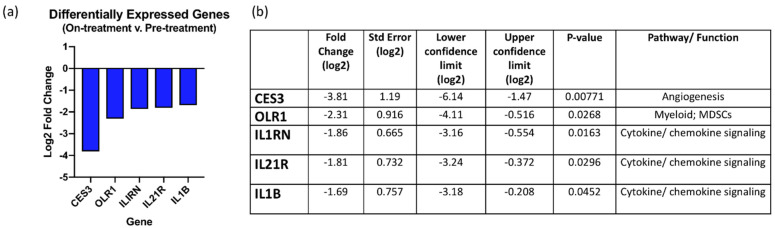
Effects of sotrastaurin and alpelisib on immune gene expression. (**a**,**b**) Gene expression analysis of immune-related genes was performed using the Nanostring IO360 Panel. Differentially expressed genes (on-treatment versus pre-treatment, n = 7 specimens per time-point) with log2 fold change >1.5 (and raw *p*-value < 0.05) are graphed and listed in the table (**b**). There were no genes demonstrating increased expression on-treatment compared to pre-treatment.

PKC isoforms have been demonstrated to regulate initiation and homeostasis of immune responses. Specifically, PKC-alpha is involved in T-cell proliferation and IFN-gamma production, and PKC-theta regulates T-cell activation and IL-2 production [17,18,19]. Given the known immunosuppressive effects of PKC and PI3K inhibition, we hypothesized that treatment with sotrastaurin and alpelisib may result in immunosuppressive effects, which may impact anti-tumor activity. To investigate this hypothesis, gene expressions for immune-related genes were analyzed on paired pre- and post-treatment tumor biopsies using the Nanostring^TM^ IO360 panel. Differentially expressed genes (on-treatment versus pre-treatment, n = 7 specimens per time-point) with log_2_ fold change >1.5 (and *p*-value < 0.05) are graphed and listed in Figure 4a,b. There were significant changes to genes involved in interferon gamma or interleukin 2 signaling, and there were no genes demonstrating significantly increased expression on-treatment compared to pre-treatment. Several genes involved in cytokine pathways were found to have decreased expression on-treatment when compared to baseline, including IL1B (IL1 beta), IL1RN (IL1 receptor antagonist), and IL21R (IL21 receptor). IL1B is a pro-inflammatory cytokine that has been shown to be associated with tumor growth and metastasis, while IL1RN is a receptor antagonist for IL1 [20]. IL21R encodes the cytokine receptor for IL21, which is predominantly produced by CD4 T cells and NK cells [21]. Expression of OLR1 (oxidized low-density lipoprotein receptor 1), which has been associated with PMN-MDSCs [22], and CES3 (carboxylesterase 3), involved in metabolism and angiogenesis, were also decreased in on-treatment relative to pre-treatment specimens.

## 4. Discussion

This was the first study to assess the therapeutic strategy of combined PKC and PI3K inhibition in patients with metastatic uveal melanoma. Our findings showed that the safety profile of sotrastaurin and alpelisib was generally acceptable, with recommended phase 2 doses of sotrastaurin 200 mg twice daily and alpelisib 350 mg daily (dose level 4). The most frequently observed AEs were GI-related, including diarrhea and nausea. Most of these were low-grade, with only 8% being Grade 3. Fatigue and anorexia were also common and predominantly low-grade. However, both GI-related symptoms and fatigue were the cause of 3 out of 5 DLTs. No clinical responses were seen with the best response of stable disease in 67% of patients. No differences in response were seen across dose levels. One patient achieved the best response of prolonged stable disease for 51 weeks.

We investigated several reasons why clinical activity was disappointing in this study, which demonstrated a similarly poor median PFS (7 versus 11 weeks) as the sotrastaurin monotherapy study that recommended a phase 2 dose of 700 mg twice daily [15]. Although the MTD for sotrastaurin in our trial was significantly lower, the concordant kinetics suggest that co-administration did not abrogate exposure to treatment and there was no significant drug–drug interaction between the two agents. Pharmacodynamic analysis on a subset of paired tumor samples showed that nearly all patients had decreased pAKT protein expression and 5 out of 7 patients with detectable pMARCKS and pS6 at baseline had a reduction on treatment, which suggests the relatively lower dose of sotrastaurin still impacted tumor signaling. Similar to the monotherapy trial, however, this effect on PKC pathway protein expression did not result in clinical benefit. Recent preliminary results from a phase 1 study of darovasertib, a potent PKC inhibitor more selectively targeting “novel” than “classical” PKC isoforms, suggest an encouraging response rate and duration of disease stability in the recommended phase 2 dose [23,24]. Further validation of this response signal would suggest that selective PKC inhibitors may be more effective than pan-PKC inhibitors such as sotrastaurin used in this study.

The limited clinical efficacy in this study unfortunately compares similarly to previous negative trials using combinations such as MEK and AKT inhibition [25], MEK inhibition combined with dacarbazine, and combined mTOR and IGF1-R inhibition [4,26]. This study adds to our knowledge regarding combination targeted therapies in uveal melanoma and suggests that rational combinations, although associated with common toxicities that can extend beyond the initial DLT period, can nonetheless be safely administered. We also utilized gene expression analysis of paired tumor samples to suggest that there was no evidence that sotrastaurin and alpelisib led to immune suppression in the tumor microenvironment. These lessons become increasingly important as additional targeted therapy trials in metastatic uveal melanoma are underway. These include combination PKC + MEK and MET inhibition with darovasertib plus binimetinib or crizotinib (NCT03947385), a phase I/II trial assessing FAK (focal adhesion kinase) and MEK inhibition (NCT04109456), and a randomized phase II trial of paclitaxel and MEK inhibition (ISRCTN 29621851) [27]. As our understanding of uveal melanoma biology increases and more clinical trials utilize targeted inhibitors, it will become increasingly important to identify rational strategies to combine antineoplastic agents.

## 5. Conclusions

In conclusion, the findings of this phase Ib study of sotrastaurin and alpelisib have demonstrated a safety profile, pharmacodynamic effects, and antitumor activity consistent with other targeted inhibitors in uveal melanoma, with key toxicities including nausea, diarrhea, fatigue, and anorexia. Given the lack of efficacy, there are no plans for development of this combination in patients with uveal melanoma. Other ongoing trials including selective PKC inhibition may offer more promise in this challenging disease.

## Figures and Tables

**Table 1:** Patient demographics.

	N (%) (Total = 25)
Age	
18–49 years old	8/25 (32%)
50–69 years old	10/25 (40%)
≥70 years old	7/25 (28%)
Median age, range	62 years, 25–78 years
Sex	
Male	16/25 (64%)
Female	9/25 (36%)
ECOG Performance Status	
0	15/25 (60%)
1	10/25 (40%)
Race	
White	25/25 (100%)
Other	0
Diagnosis	
Uveal Melanoma	24/25 (96%)
Melanoma unknown primary (GNAQ/11 mutant)	1/25 (4%)
GNAQ or GNA11 mutation status	
GNAQ mutant	14/25 (56%)
GNA11 mutant	8/25 (32%)
Wildtype	3/25 (12%)
Uveal AJCC M stage	
M1a	6/25 (24%)
M1b	7/25 (28%)
M1c	8/25 (32%)
Unknown	3/25 (12%)
Not applicable (non-Uveal)	1/25 (4%)
Prior Metastatic Treatments, #	
Median	3
Range	0–8
LDH at baseline	
Elevated	11/25 (44%)
Normal	6/25 (24%)
Unknown	8/25 (32%)
Alkaline Phosphatase at baseline	
Elevated	13/25 (52%)
Normal	12/25 (48%)

# denotes the word number.

**Table 2:** Dose-limiting toxicities by dose level.

	n, Total Patients in Dose Level	n, # DLTs	DLT Information
Dose Level 1	9	2	• Grade 3 hyperglycemia (n = 1) • Grade 3 nausea (n = 1)
Dose Level 2	3	0	
Dose Level 3	3	0	
Dose Level 4	6	1	• Intolerable Grade 2 fatigue (n = 1)
Dose Level 5	3	2	• Intolerable Grade 2 fatigue, diarrhea, nausea (n = 1) • Grade 3 neutropenia > 7 days (n = 1)
All	24	5	

# denotes the word number.

**Table 3:** Adverse events (all grades, occurring in 5% of patients) suspected to be related to study treatment.

	All Patients (N = 25)				
	Grade 1 n (%)	Grade 2 n (%)	Grade 3 n (%)	Grade 4–5 n (%)	All Grades
Laboratory					
Hyperglycemia	7 (28%)	1 (4%)	2 (8%)	0	10 (40%)
Anemia	3 (12%)	2 (8%)	0	0	5 (20%)
Alkaline Phos increased	2 (8%)	2 (8%)	0	0	4 (16%)
ALT increased	2 (8%)	0	1 (4%)	0	3 (12%)
AST increased	1 (4%)	0	1 (4%)	0	2 (8%)
Bilirubin increased	2 (8%)	0	0	0	2 (8%)
Neutrophil count decreased	0	0	1 (4%)	0	1 (4%)
Gastrointestinal					
Nausea	3 (12%)	11 (44%)	2 (8%)	0	16 (64%)
Diarrhea	11 (44%)	2 (8%)	2 (8%)	0	15 (60%)
Anorexia	4 (16%)	5 (20%)	1 (4%)	0	10 (40%)
Vomiting	5 (20%)	1 (4%)	1 (4%)	0	7 (28%)
Dysgeusia	1 (4%)	2 (8%)	0	0	3 (12%)
Dehydration	0	0	1 (4%)	0	1 (4%)
Other					
Fatigue	2 (8%)	9 (36%)	2 (8%)	0	13 (52%)
Acneiform rash	4 (16%)	1 (4%)	0	0	5 (20%)
Malaise	3 (12%)	1 (4%)	0	0	4 (16%)

**Table 4:** RECIST responses by dose level.

	Level 1	Level 2	Level 3	Level 4	Level 5	All Levels
Complete Response (CR)	0	0	0	0	0	0
Partial Response (PR)	0	0	0	0	0	0
Stable Disease (SD)	4/9 (44.4%)	3/3 (100%)	2/3 (66.7%)	4/6 (66.7%)	3/3 (100%)	16/24 (66.7%)
Progressive Disease (PD)	5/9 (55.6%)	0	1/3 (33.3%)	2/6 (33.3%)	0	8/24 (33.3%)
Overall Response Rate (ORR)	0	0	0	0	0	0
Median Time to Progression (range), weeks	5.5 weeks (3–16.4)	30.1 weeks (8–51)	10.8 weeks (3.4–16)	15.8 weeks (4–23.7)	14 weeks (11.4–16)	Mean: 12.8 weeks, Median 7.6 weeks

**Table 5:** Mean pre-dose plasma concentration over time, ng/mL (SD).

	Cycle 1	Cycle 2	Cycle 3	Cycle 4	Cycle 5	Cycle 6
Sotrastaurin						
Level 1	0	271.9 (138.3)				
Level 2	0	404.0 (0)	593.0 (0)	720.0 (0)	614.0 (0)	631.0 (0)
Level 3	0	640.0 (905.1)	675.0 (741.8)	458.0 (454.0)	612.0 (0)	
Level 4	0	2180.0 (0)	2380.0 (0)			
Level 5	0	729.0 (0)	475.5 (140.7)	861.0 (0)		
Alpelisib						
Level 1	0	132.1 (62.7)				
Level 2	0	103.0 (0)	180.0 (0)	147.0 (0)	162.0 (0)	137.0 (0)
Level 3	0	101.5 (143.5)	204.0 (97.6)	156.5 (53.0)	225.0 (0)	
Level 4	0	364.0 (0)	497.0 (0)			
Level 5	0	129.0 (182.4)	431.3 (554.0)	41.6 (0)		

## Data Availability

The data presented in this study are available upon request from the corresponding author.

## Supplementary Materials

The following are available online at https://www.mdpi.com/article/10.3390/cancers13215504/s1, File S1: original blots.

## Appendix A

**Table A1:** Dose levels.

Dose Level	Sotrastaurin	Alpelisib
Level −1	100 mg BID	150 mg daily
Level 1	100 mg BID	200 mg daily
Level 2	200 mg BID	250 mg daily
Level 3	200 mg BID	300 mg daily
Level 4	200 mg BID	350 mg daily
Level 5	300 mg BID	350 mg daily
Level 6	400 mg BID	350 mg daily

**Table A2:** Baseline characteristics for patients achieving stable disease > 20 weeks.

Identifier	Dose Level	Age	Sex	Sites of Metastatic Disease	Prior Systemic Therapies
001-09	2	47	M	Omentum	None; resection × 2
002-04	2	74	M	heart, mediastinal LN, bone	(1) Selumetinib + DTIC, (2) Ipilimumab, (3) Pembrolizumab
003-02	4	49	F	Liver	None

**Table A3:** Summary of pharmacokinetic parameters.

	Half-Life (Mean)		Time to Maximum Concentration (Mean)		Maximum Concentration (Mean)		Area Under the Curve (Mean)	
	Day 1 (hours)	Day 8 (hours)	Day 1 (hours)	Day 8 (hours)	Day 1 (ng/mL)	Day 8 (ng/mL)	Day 1 (μg × h/mL)	Day 8 (μg × h/mL)
Sotrastaurin								
Level 1 (n = 7)	4.62	5.26	2.33	1.0	859.56	1209.29	4259.08	5932.04
Level 2 (n = 11)	5.90	4.82	2.33	1.27	1673.17	1848.82	6857.01	8766.88
Level 3 (n = 1)	8.15	6.98	1.0	2.0	2217.67	3600.00	12,513.19	19,210.00
Alpelisib								
Level 1 (n = 7)	6.44	7.60	5.14	3.28	915.43	1002.14	11,163.35	13,105.43
Level 2 (n = 1)	2.43	5.46	4.0	4.0	1390.00	1900.00	7064.25	18,265.25
Level 3 (n = 2)	7.87	4.59	4.0	4.0	1163.50	1820.00	16,203.75	19,726.79
Level 4 (n = 4)	10.69	11.08	6.5	4.67	1462.50	1530.00	21,862.81	23,726.42

**Table A4:** Clinical summary of patients with samples analyzed for PD, gene expression, and mIHC.

Sample ID	Dose Level	GNAQ/11 Status	Best Response (sum of TL)	Time to Progression	Western Analysis	Gene Expression
001-13	Level 4	GNA11 Q209L	SD (+14%)	7.1 weeks	X	X
001-17	Level 4	GNAQ Q209L	SD (+4%)	8.5 weeks	X	X
002-10	Level 4	GNAQ Q209P	SD (−11%)	8.0 weeks	X	X
003-01	Level 4	GNA11 Q209L	PD (−19% *)	4.0 weeks	X	X
003-04	Level 4	GNAQ Q209L	PD (+10.7% **)	4.0 weeks	X	X
001-15	Level 5	GNAQ	SD (0%)	14.6 weeks	X	X
001-16	Level 5	GNAQ Q209P	SD (0%)	11.4 weeks	X	X

TL = target lesion; SD = stable disease; PD = progressive disease; * = PD due to progression in non-target lesion; ** = PD due to new lesion.

## References

[1] Singh A.D., Turell M.E., Topham A.K. (2011). Uveal melanoma: Trends in incidence, treatment, and survival. Ophthalmology.

[2] Chang A.E., Karnell L.H., Menck H.R. (1998). The National Cancer Data Base report on cutaneous and noncutaneous melanoma: A summary of 84,836 cases from the past decade. The American College of Surgeons Commission on Cancer and the American Cancer Society. Cancer.

[3] Piperno-Neumann S., Hassel J.C., Rutkowski P., Baurain J.-F., Butler M.O., Schlaak M., Sullivan R.J., Ochsenreither S., Dummer R., Kirkwood J.M. (2021). Abstract CT002: Phase 3 randomized trial comparing tebentafusp with investigator’s choice in first line metastatic uveal melanoma. J. Cancer Res..

[4] Carvajal R.D., Piperno-Neumann S., Kapiteijn E., Chapman P.B., Frank S., Joshua A.M., Piulats J.M., Wolter P., Cocquyt V., Chmielowski B. (2018). Selumetinib in Combination With Dacarbazine in Patients With Metastatic Uveal Melanoma: A Phase III, Multicenter, Randomized Trial (SUMIT). J. Clin. Oncol..

[5] Van Raamsdonk C.D., Bezrookove V., Green G., Bauer J., Gaugler L., O’Brien J.M., Simpson E.M., Barsh G.S., Bastian B.C. (2009). Frequent somatic mutations of GNAQ in uveal melanoma and blue naevi. Nature.

[6] Van Raamsdonk C.D., Griewank K.G., Crosby M.B., Garrido M.C., Vemula S., Wiesner T., Obenauf A.C., Wackernagel W., Green G., Bouvier N. (2010). Mutations in GNA11 in uveal melanoma. N. Engl. J. Med..

[7] Rozengurt E. (2007). Mitogenic signaling pathways induced by G protein-coupled receptors. J. Cell. Physiol..

[8] Lee C.H., Park D., Wu D., Rhee S.G., Simon M.I. (1992). Members of the Gq alpha subunit gene family activate phospholipase C beta isozymes. J. Biol. Chem..

[9] Koivunen J., Aaltonen V., Peltonen J. (2006). Protein kinase C (PKC) family in cancer progression. Cancer Lett..

[10] Wu X., Zhu M., Fletcher J.A., Giobbie-Hurder A., Hodi F.S. (2012). The protein kinase C inhibitor enzastaurin exhibits antitumor activity against uveal melanoma. PLoS ONE.

[11] Wu X., Li J., Zhu M., Fletcher J.A., Hodi F.S. (2012). Protein kinase C inhibitor AEB071 targets ocular melanoma harboring GNAQ mutations via effects on the PKC/Erk1/2 and PKC/NF-kappaB pathways. Mol. Cancer Ther..

[12] Piperno-Neumann S., Kapiteijn E., Larkin J.M.G., Carvajal R.D., Luke J.J., Seifert H., Roozen I., Zoubir M., Yang L., Choudhury S. (2014). Phase I dose-escalation study of the protein kinase C (PKC) inhibitor AEB071 in patients with metastatic uveal melanoma. J. Clin. Oncol..

[13] Musi E., Ambrosini G., de Stanchina E., Schwartz G.K. (2014). The phosphoinositide 3-kinase alpha selective inhibitor BYL719 enhances the effect of the protein kinase C inhibitor AEB071 in GNAQ/GNA11-mutant uveal melanoma cells. Mol. Cancer Ther..

[14] Tomfohr J., Lu J., Kepler T.B. (2005). Pathway level analysis of gene expression using singular value decomposition. BMC Bioinformatics.

[15] Piperno-Neumann S., Larkin J., Carvajal R.D., Luke J.J., Schwartz G.K., Hodi F.S., Sablin M.P., Shoushtari A.N., Szpakowski S., Chowdhury N.R. (2020). Genomic Profiling of Metastatic Uveal Melanoma and Clinical Results of a Phase I Study of the Protein Kinase C Inhibitor AEB071. Mol. Cancer Ther..

[16] Herget T., Oehrlein S.A., Pappin D.J., Rozengurt E., Parker P.J. (1995). The myristoylated alanine-rich C-kinase substrate (MARCKS) is sequentially phosphorylated by conventional, novel and atypical isotypes of protein kinase C. Eur. J. Biochem..

[17] Zhang E.Y., Kong K.F., Altman A. (2013). The yin and yang of protein kinase C-theta (PKCtheta): A novel drug target for selective immunosuppression. Adv. Pharmacol..

[18] Kovarik J.M., Slade A. (2010). Overview of sotrastaurin clinical pharmacokinetics. Ther. Drug Monit..

[19] Pfeifhofer-Obermair C., Thuille N., Baier G. (2012). Involvement of distinct PKC gene products in T cell functions. Front. Immunol..

[20] Guo B., Fu S., Zhang J., Liu B., Li Z. (2016). Targeting inflammasome/IL-1 pathways for cancer immunotherapy. Sci. Rep..

[21] Davis M.R., Zhu Z., Hansen D.M., Bai Q., Fang Y. (2015). The role of IL-21 in immunity and cancer. Cancer Lett..

[22] Condamine T., Dominguez G.A., Youn J.I., Kossenkov A.V., Mony S., Alicea-Torres K., Tcyganov E., Hashimoto A., Nefedova Y., Lin C. (2016). Lectin-type oxidized LDL receptor-1 distinguishes population of human polymorphonuclear myeloid-derived suppressor cells in cancer patients. Sci. Immunol..

[23] Kapiteijn E., Carlino M., Boni V., Loirat D., Speetjens F., Park J., Calvo E., Carvajal R., Nyakas M., Gonzalez-Maffe J. (2019). Abstract CT068: A Phase I trial of LXS196, a novel PKC inhibitor for metastatic uveal melanoma. Cancer Res..

[24] (2020). A phase 1/2 study of IDE196 in patients with metastatic uveal melanoma or solid tumors harboring GNAQ/11 mutations or PRKC fusions. Pigment. Cell Melanoma. Res..

[25] Shoushtari A.N., Kudchadkar R.R., Panageas K., Murthy R.K., Jung M., Shah R., O’Donnell B., Khawaja T.T., Shames Y., Prempeh-Keteku N.A. (2016). A randomized phase 2 study of trametinib with or without GSK2141795 in patients with advanced uveal melanoma. J. Clin. Oncol..

[26] Shoushtari A.N., Ong L.T., Schoder H., Singh-Kandah S., Abbate K.T., Postow M.A., Callahan M.K., Wolchok J., Chapman P.B., Panageas K.S. (2016). A phase 2 trial of everolimus and pasireotide long-acting release in patients with metastatic uveal melanoma. Melanoma Res..

[27] Nathan P., Needham A., Corrie P.G., Danson S., Evans J., Ochsenreither S., Kumar S., Goodman A., Larkin J.M.G., Karydis I. (2019). LBA73-SELPAC: A 3 arm randomised phase II study of the MEK inhibitor selumetinib alone or in combination with paclitaxel (PT) in metastatic uveal melanoma (UM). Ann. Oncol..

