# Correction: Shoushtari et al. A Phase Ib Study of Sotrastaurin, a PKC Inhibitor, and Alpelisib, a PI3Kα Inhibitor, in Patients with Metastatic Uveal Melanoma. *Cancers* 2021, *13*, 5504

**DOI:** 10.3390/cancers17183018

**Published:** 2025-09-16

**Authors:** Alexander N. Shoushtari, Shaheer Khan, Kimberly Komatsubara, Lynn Feun, Nicolas Acquavella, Shahnaz Singh-Kandah, Tiffany Negri, Alexandra Nesson, Kelly Abbate, Serge Cremers, Elgilda Musi, Grazia Ambrosini, Shing Lee, Gary K. Schwartz, Richard D. Carvajal

**Affiliations:** 1 Memorial Sloan Kettering Cancer Center, New York, NY 10065, USA; abbatek@mskcc.org; 2 Columbia University Irving Medical Center, New York, NY 10032, USA; sk4488@cumc.columbia.edu (S.K.); kmkomatsubara@gmail.com (K.K.); svs2126@cumc.columbia.edu (S.S.-K.); tn2280@cumc.columbia.edu (T.N.); an2843@cumc.columbia.edu (A.N.); sc2752@cumc.columbia.edu (S.C.); em3058@cumc.columbia.edu (E.M.); ga2391@cumc.columbia.edu (G.A.); gks2123@cumc.columbia.edu (G.K.S.); rdc2150@cumc.columbia.edu (R.D.C.); 3 Sylvester Comprehensive Cancer Center, University of Miami, Miami, FL 33136, USA; lfeun@med.miami.edu (L.F.); nacquavella@miami.edu (N.A.); 4 Department of Biostatistics, Mailman School of Public Health, Columbia University, New York, NY 10032, USA; sml2114@cumc.columbia.edu

**Figure**

In the original publication [1], there was a mistake in Figure 3 in which an incorrect Western blot was inadvertently inserted under subject 001-17. Source files were reviewed, and the correct Western blot was identified. The correct Figure 3 appears below. The authors apologize for any inconvenience caused and state that the scientific conclusions are unaffected. This correction was approved by the Academic Editor. The original publication has also been updated.

## Figures and Tables

**Figure 3:**
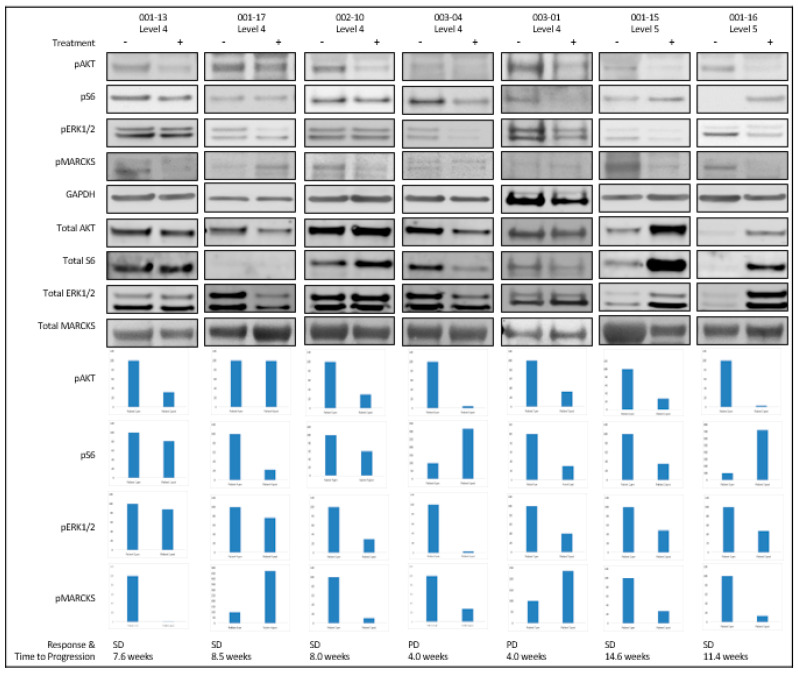
Western blot analysis of PKC and PI3Kα-AKT-mTOR pathway inhibition. Paired tumor biopsies were assessed by Western blot for pAKT, pS6, pERK1/2, pMARCKS, and the respective total proteins. GAPDH was used as a loading control. Clinical outcomes for these respective patients are listed. Protein quantitation of the pre-treatment (left) and post-treatment (right) Western blot analysis performed with Image J. The pre-treatment sample expression level represents a baseline of 100%, with the post-treatment sample expression levels relative to this baseline. Bar plots represent pAKT/total AKT, pS6/total S6, pERK1/2/ total ERK1/2, and pMARCKS/MARCKS, respectively.
