# Supplementary material for: A Phase Ib Study of Sotrastaurin, a PKC Inhibitor, and Alpelisib, a PI3Kα Inhibitor, in Patients with Metastatic Uveal Melanoma
Source: Cancers (Basel). 2021 Nov 2;13(21):5504. doi: 10.3390/cancers13215504 (PMC8583628; doi:10.3390/cancers13215504)
Supplement: Supplementary file 1 [file cancers-13-05504-s001.zip › orginal blots/Blots for 001-13, 001-15,001-16,001-17,002-10,003-004/pERK PDF.pdf]

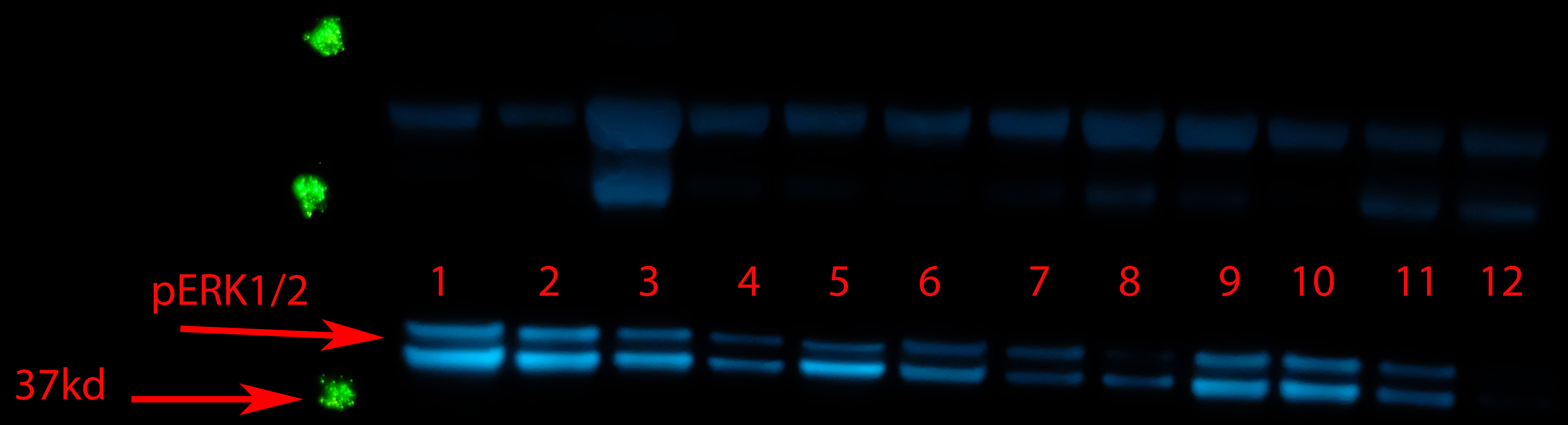

Lane 1 Patient 1 pre 001-13
Lane 2 Patient 1 post 001-13
Lane 3 Patient 2 pre 001-15
Lane 4 Patient 2 post 001-15
Lane 5 Patient 3 pre 001-16
Lane 6 Patient 3 post 001-16
Lane 7 Patient 4 pre 001-17
Lane 8 Patient 4 post 001-17
Lane 9 Patient 5 pre 002-10
Lane 10 Patient 5 post 002-10
Lane 11 Patient 6 pre 003-004
Lane 12 Patient 6 post 003-004
