# Supplementary material for: A Phase Ib Study of Sotrastaurin, a PKC Inhibitor, and Alpelisib, a PI3Kα Inhibitor, in Patients with Metastatic Uveal Melanoma
Source: Cancers (Basel). 2021 Nov 2;13(21):5504. doi: 10.3390/cancers13215504 (PMC8583628; doi:10.3390/cancers13215504)
Supplement: Supplementary file 1 [file cancers-13-05504-s001.zip › orginal blots/Blots for 001-13, 001-15,001-16,001-17,002-10,003-004/ps6 PDF.pdf]

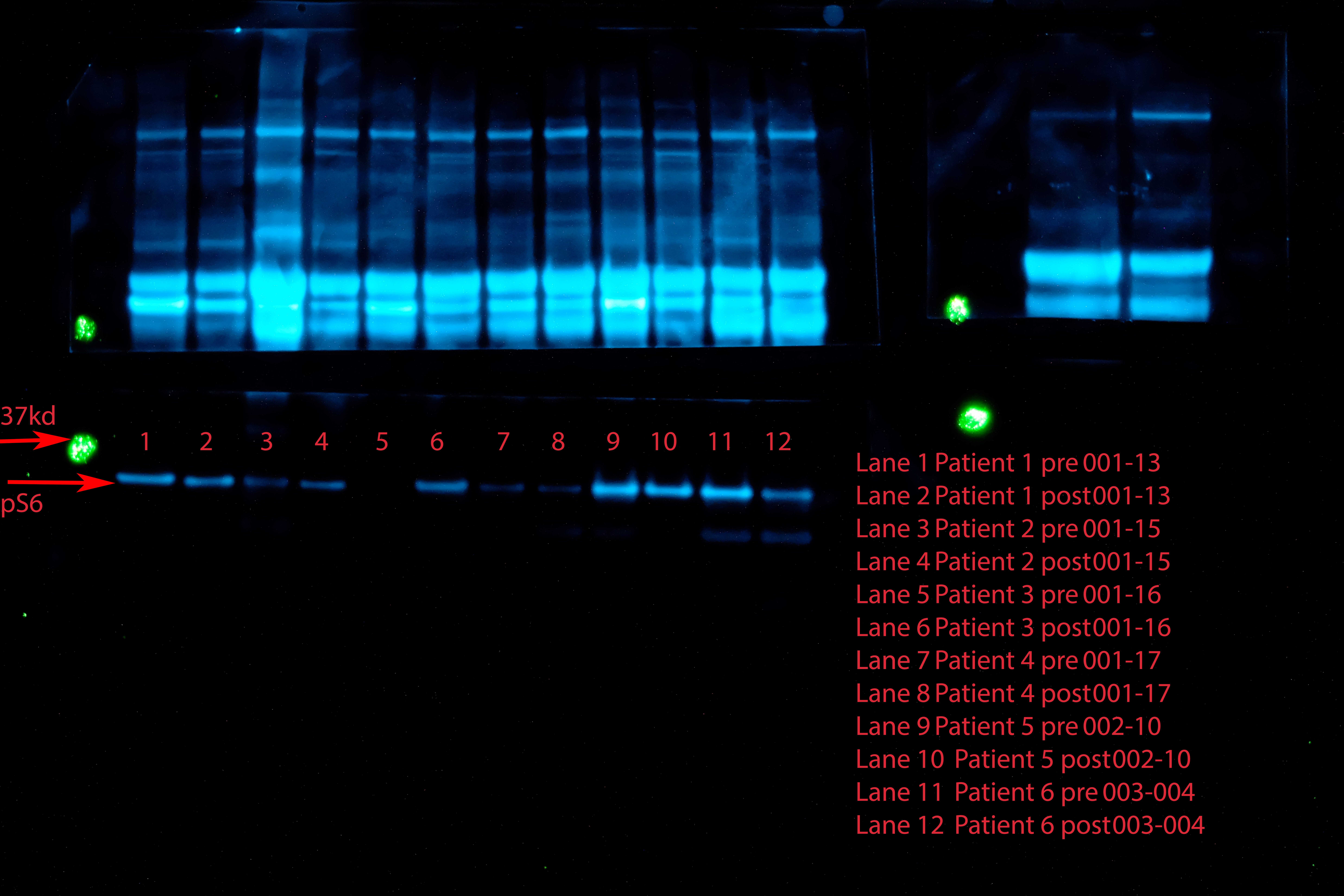
37kd
pS6
1 2 3 4 5 6 7 8 9 10 11 12
Lane 1 Patient 1 pre 001-13
Lane 2 Patient 1 post001-13
Lane 3 Patient 2 pre 001-15
Lane 4 Patient 2 post001-15
Lane 5 Patient 3 pre 001-16
Lane 6 Patient 3 post001-16
Lane 7 Patient 4 pre 001-17
Lane 8 Patient 4 post001-17
Lane 9 Patient 5 pre 002-10
Lane 10 Patient 5 post002-10
Lane 11 Patient 6 pre 003-004
Lane 12 Patient 6 post003-004
