# Supplementary figures and images for: A Phase Ib Study of Sotrastaurin, a PKC Inhibitor, and Alpelisib, a PI3Kα Inhibitor, in Patients with Metastatic Uveal Melanoma
Source: Cancers (Basel). 2021 Nov 2;13(21):5504. doi: 10.3390/cancers13215504 (PMC8583628; doi:10.3390/cancers13215504)

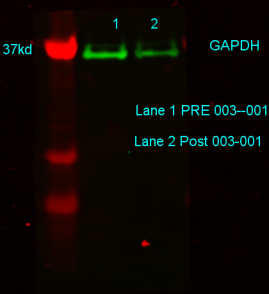

Supplement: Supplementary file 1 [file cancers-13-05504-s001.zip › orginal blots/Blots 003-001/GAPDH patient 003-001.pdf]

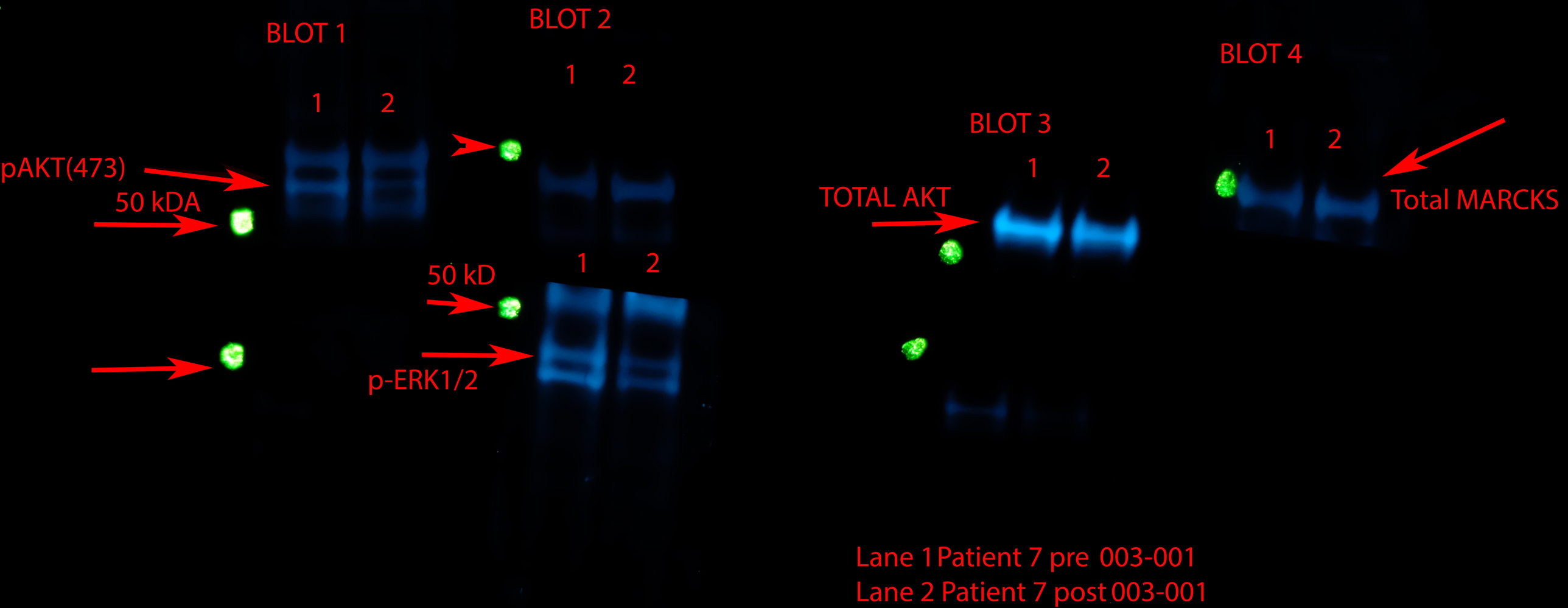

Supplement: Supplementary file 1 [file cancers-13-05504-s001.zip › orginal blots/Blots 003-001/pakt perk Total AKT Total MArcksblot for 003-001.pdf]

Blot 1

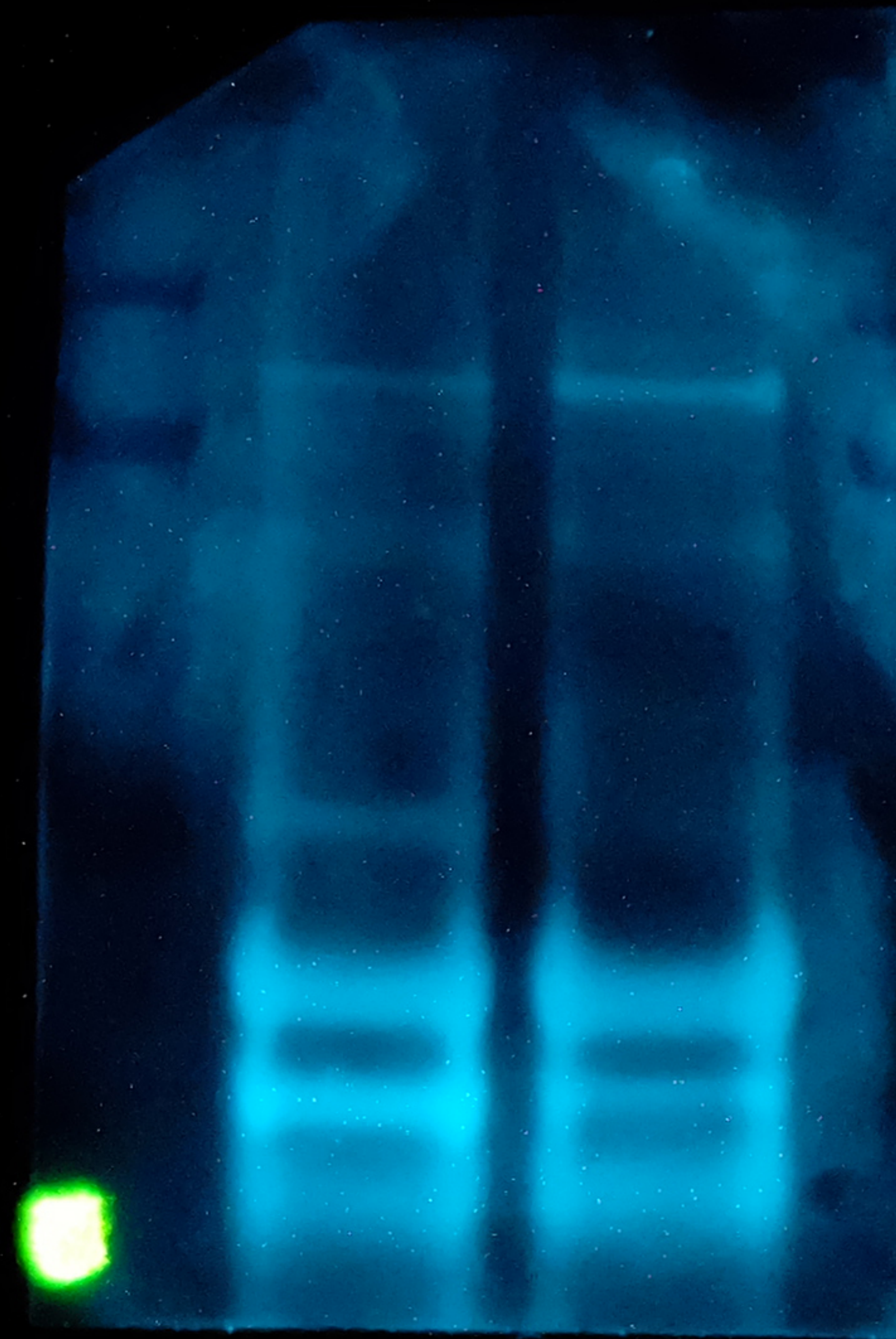

Blot 2

1 2

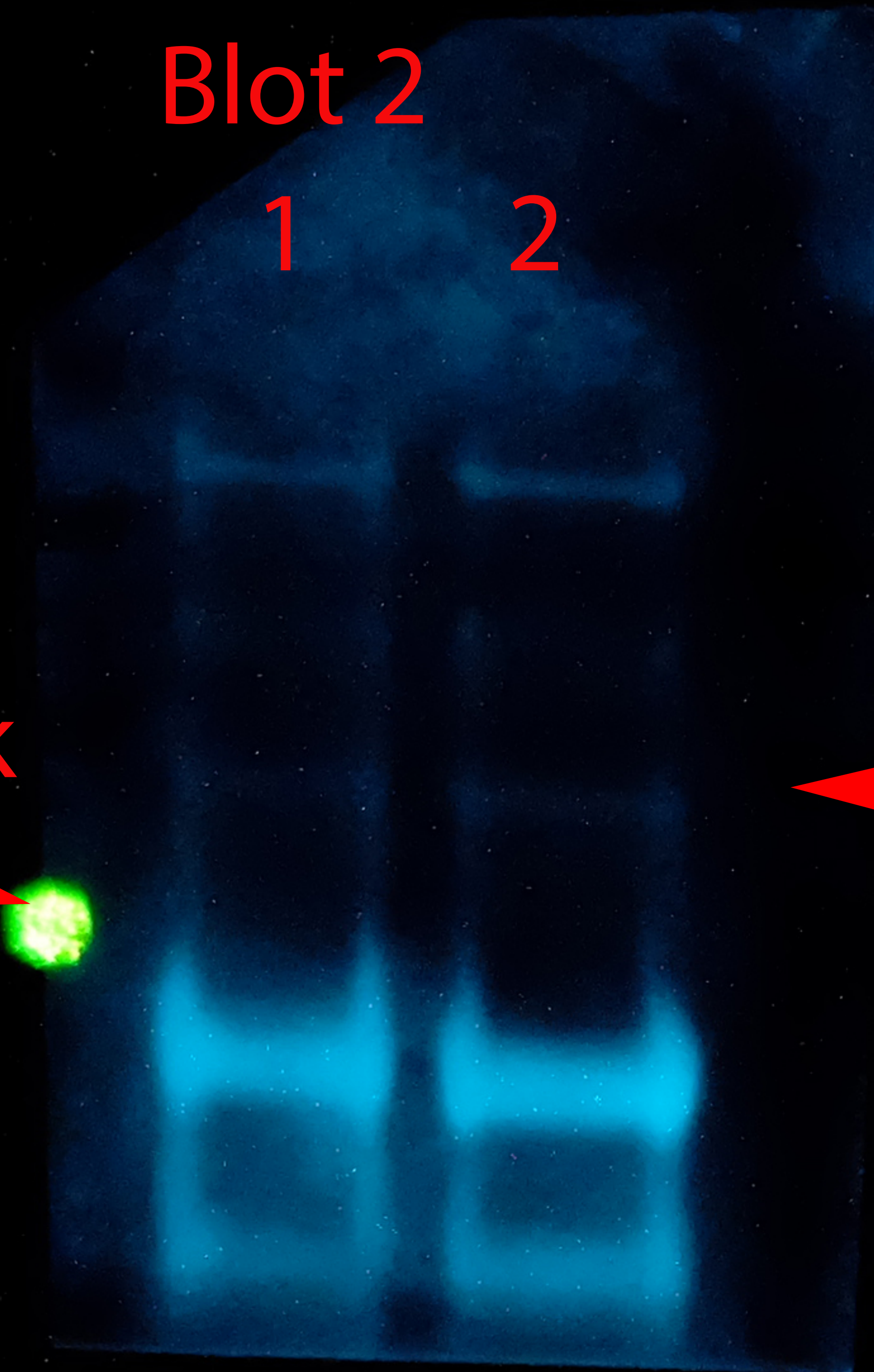

75k

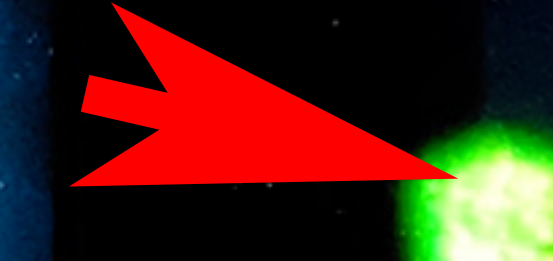

pMARCKS

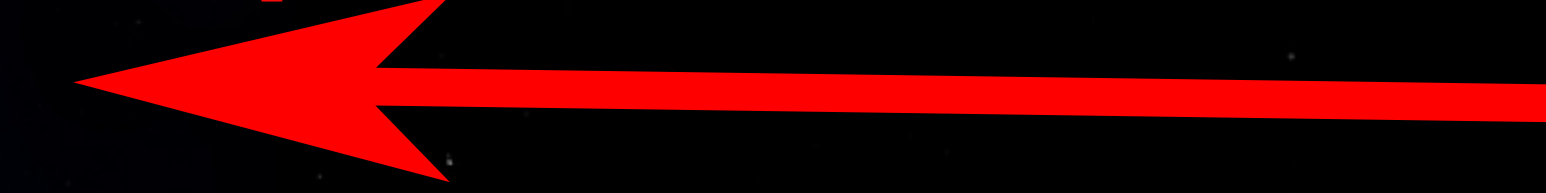

BLOT 3

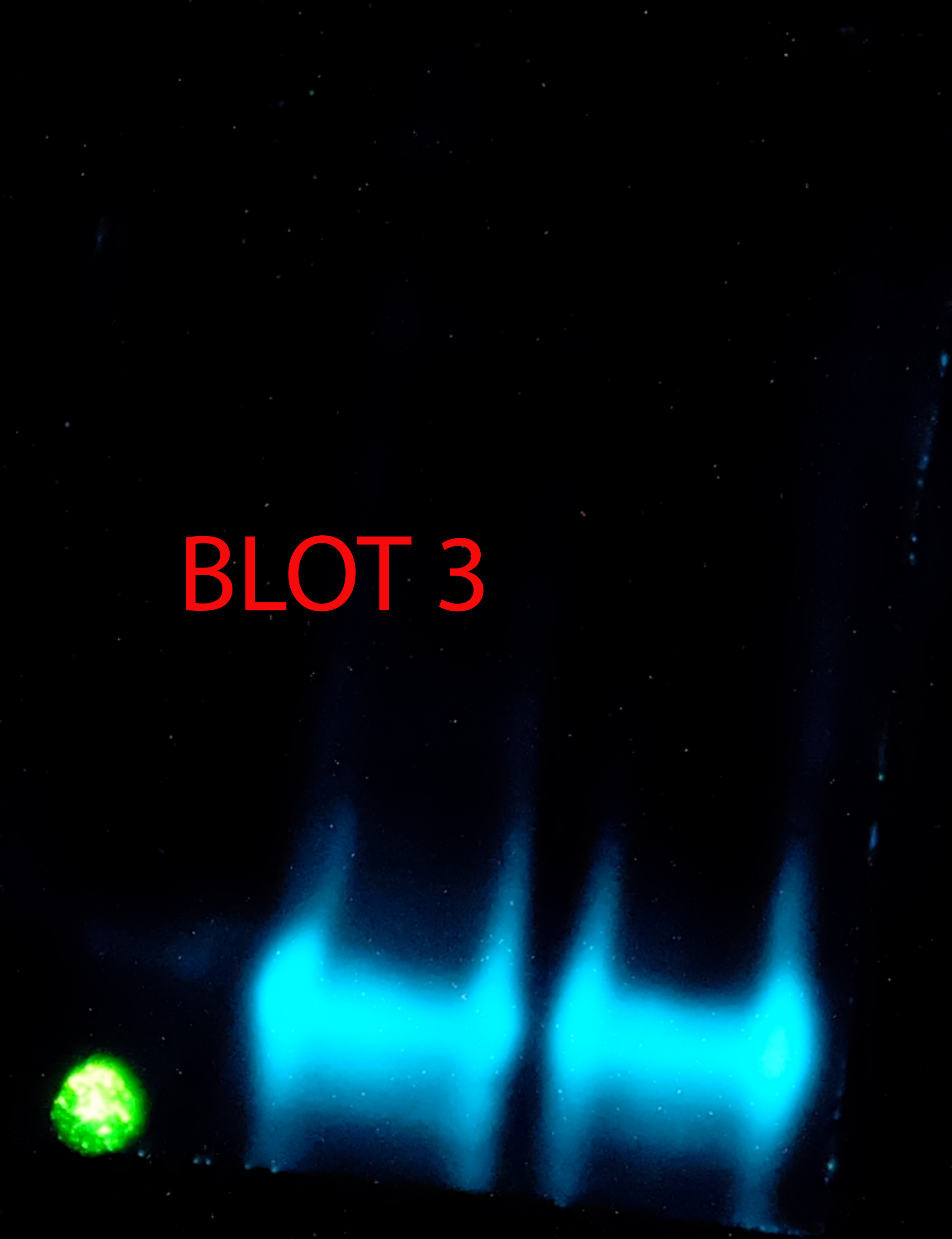

BLOT 4

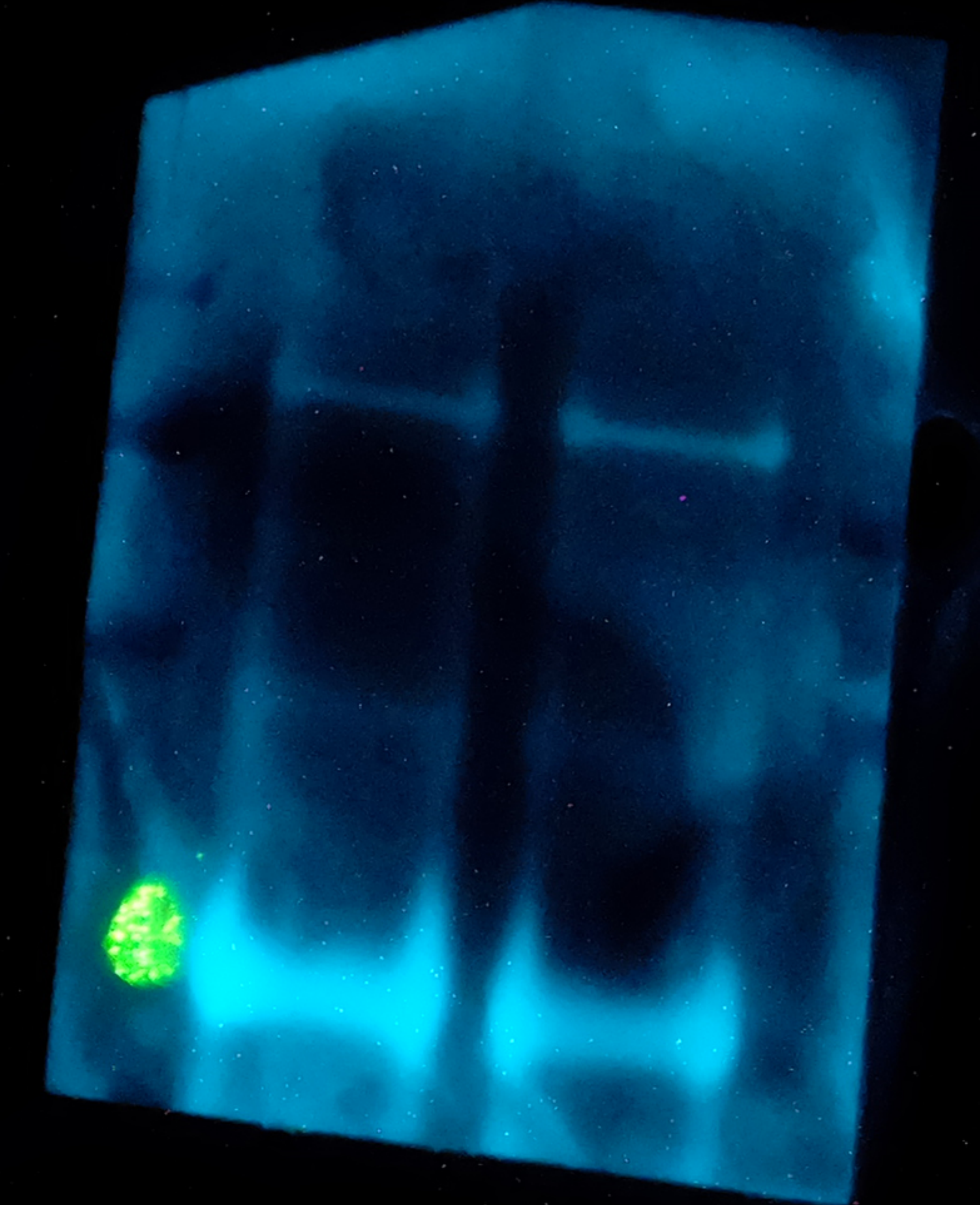

37kd

pS6

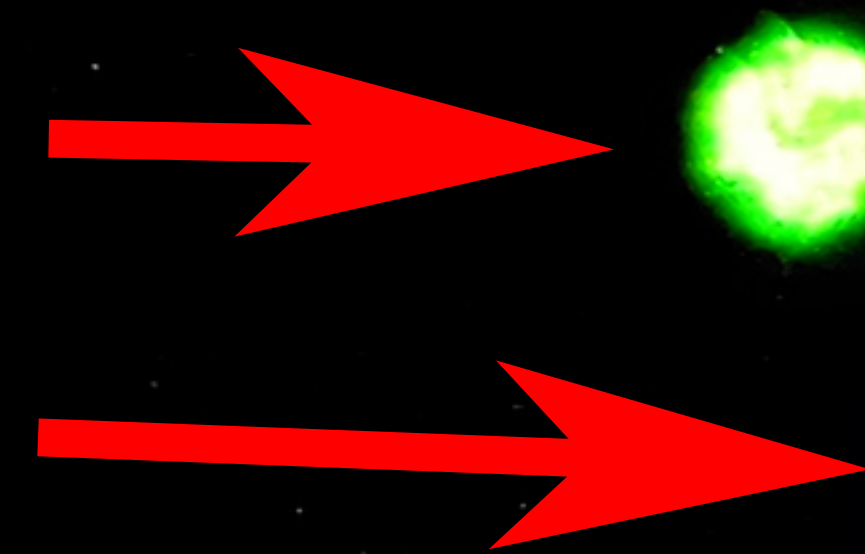

1 2

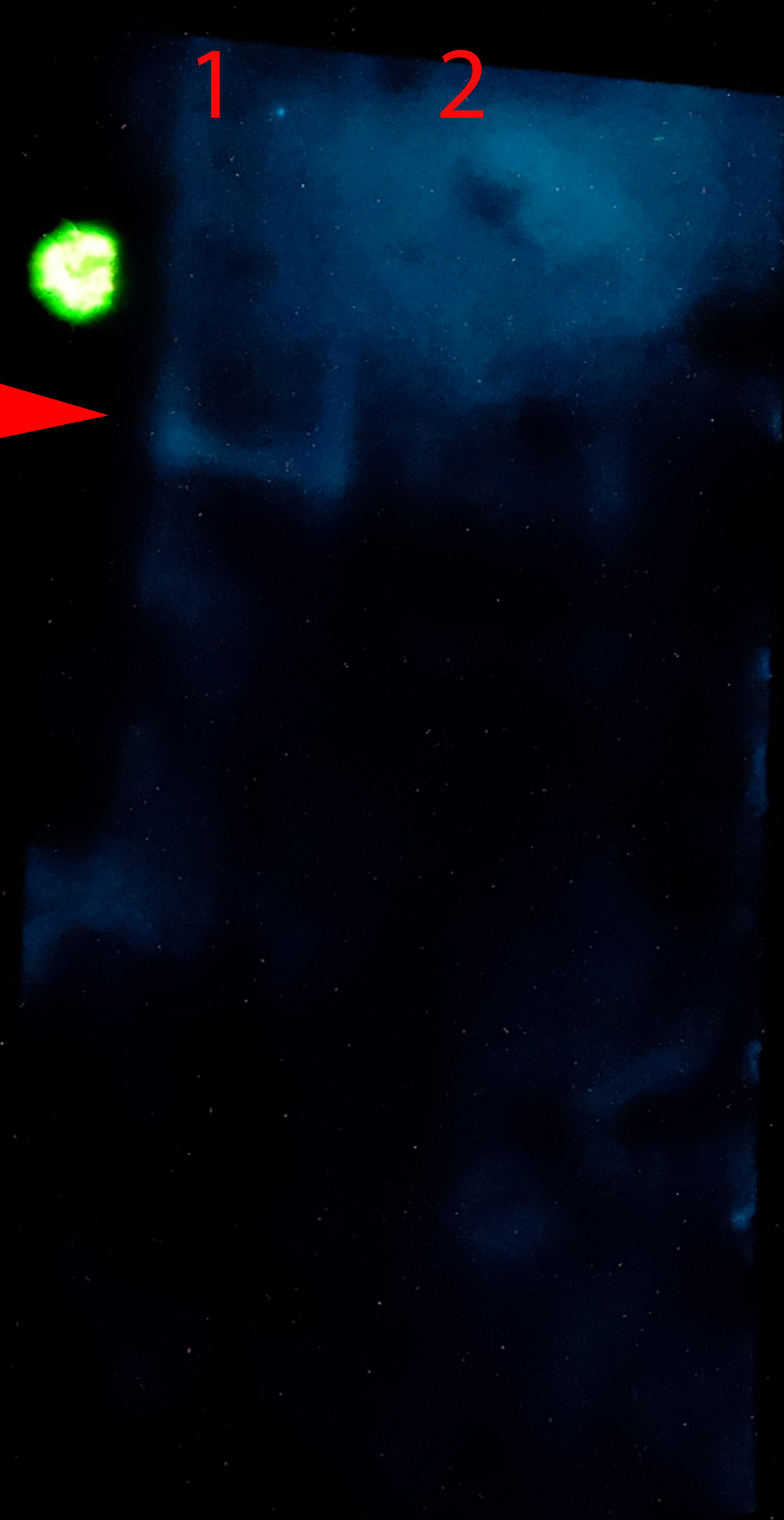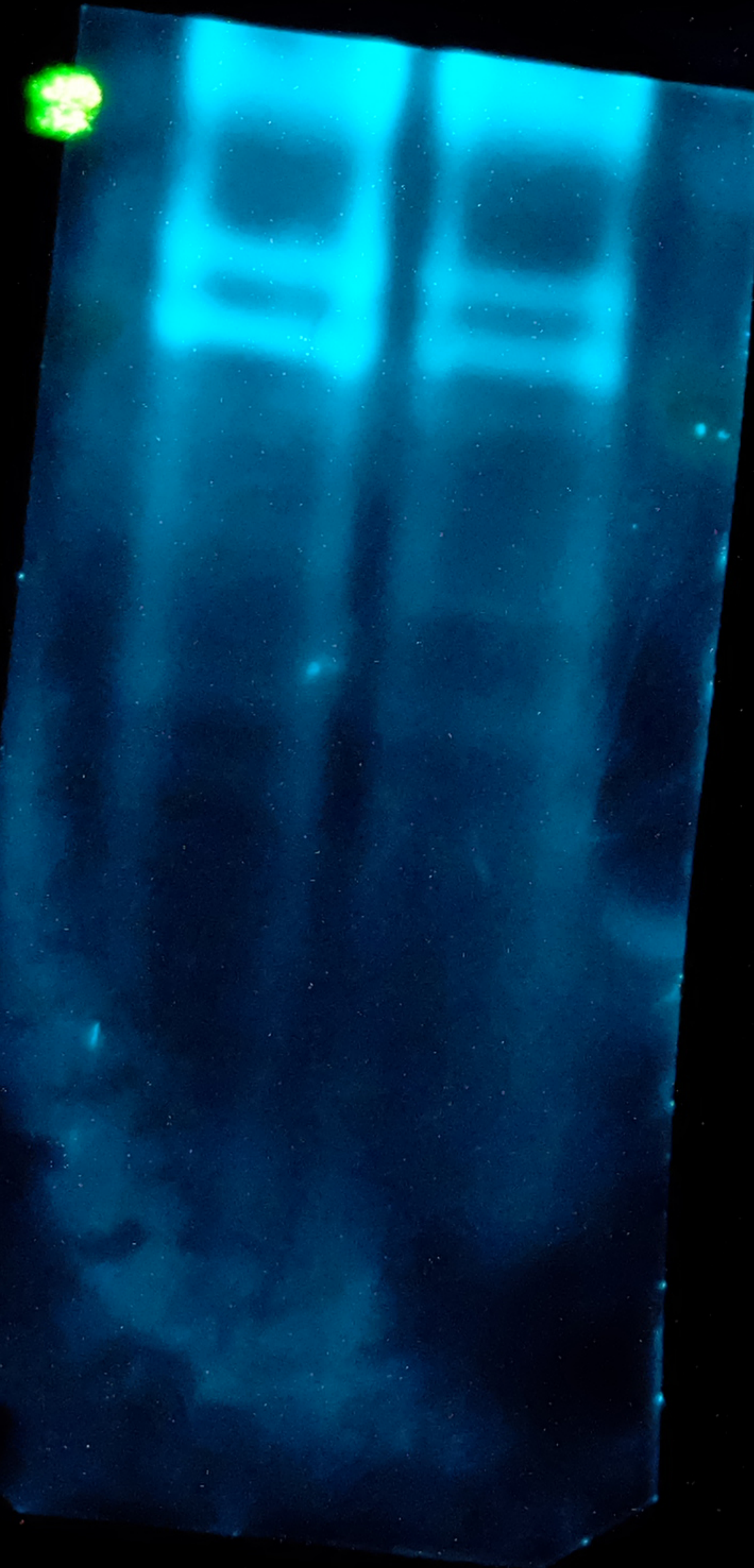

37kd

Total S6

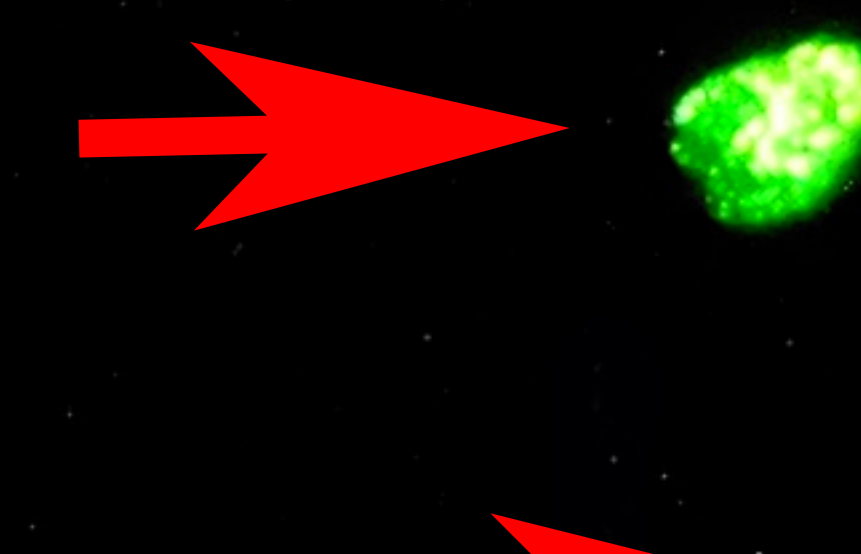

1 2

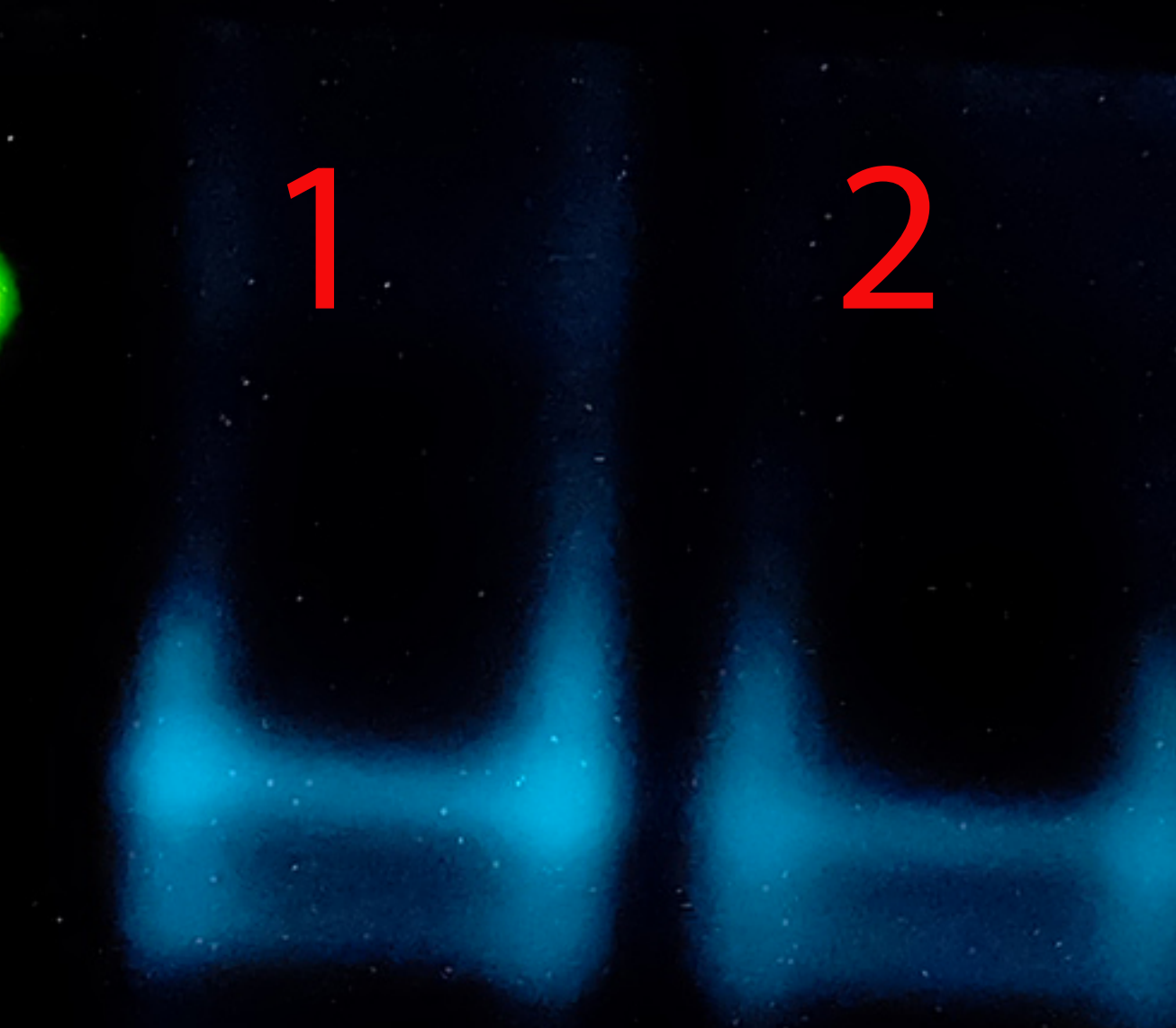

Lane 1 Patient 7 pre 003-001  
Lane 2 Patient 7 post 003-001

Supplement: Supplementary file 1 [file cancers-13-05504-s001.zip › orginal blots/Blots 003-001/pS6 total s6 and pmarcks blot for 003-001.pdf]

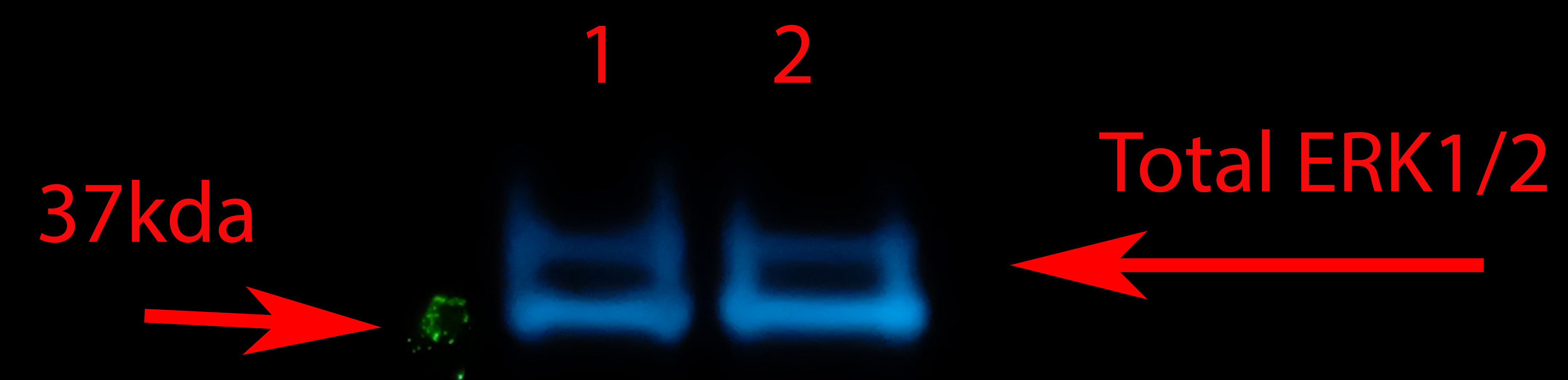

Lane 1 Patient 7 pre 003-001  
Lane 2 Patient 7 post 003-001

Supplement: Supplementary file 1 [file cancers-13-05504-s001.zip › orginal blots/Blots 003-001/TOTAL ERK12 blot for 003-001.pdf]

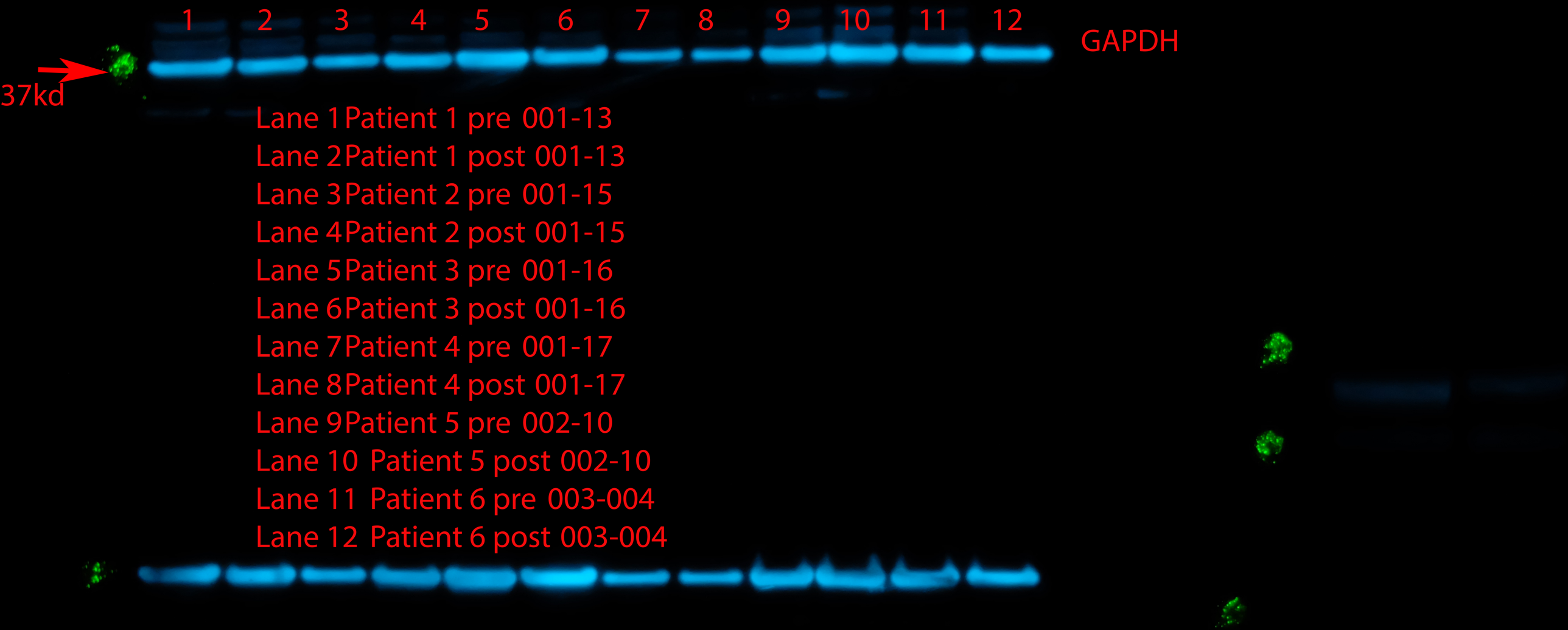

Supplement: Supplementary file 1 [file cancers-13-05504-s001.zip › orginal blots/Blots for 001-13, 001-15,001-16,001-17,002-10,003-004/GAPDH PDF.pdf]

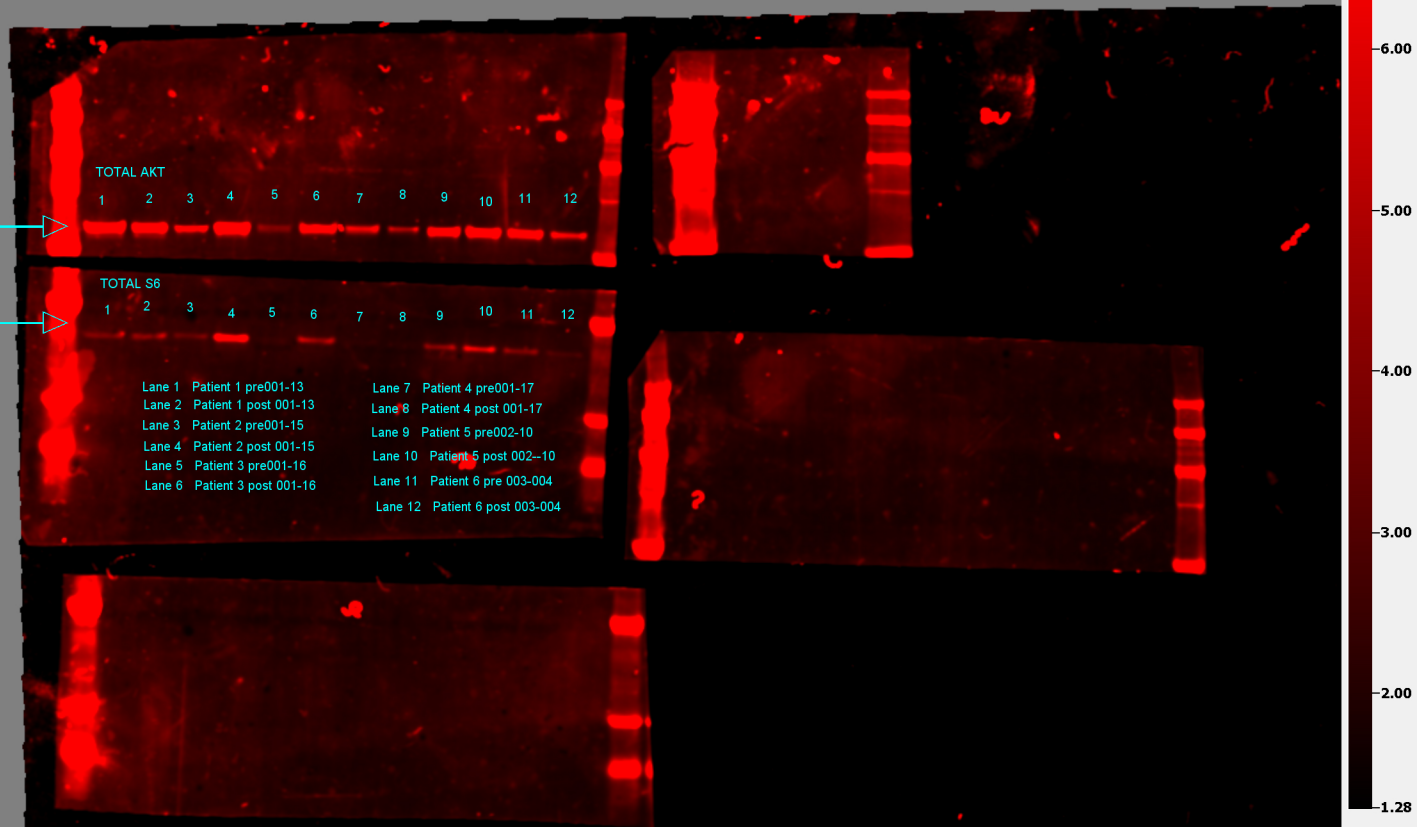

Supplement: Supplementary file 1 [file cancers-13-05504-s001.zip › orginal blots/Blots for 001-13, 001-15,001-16,001-17,002-10,003-004/Total AKT and Total S6 biopsies.pdf]

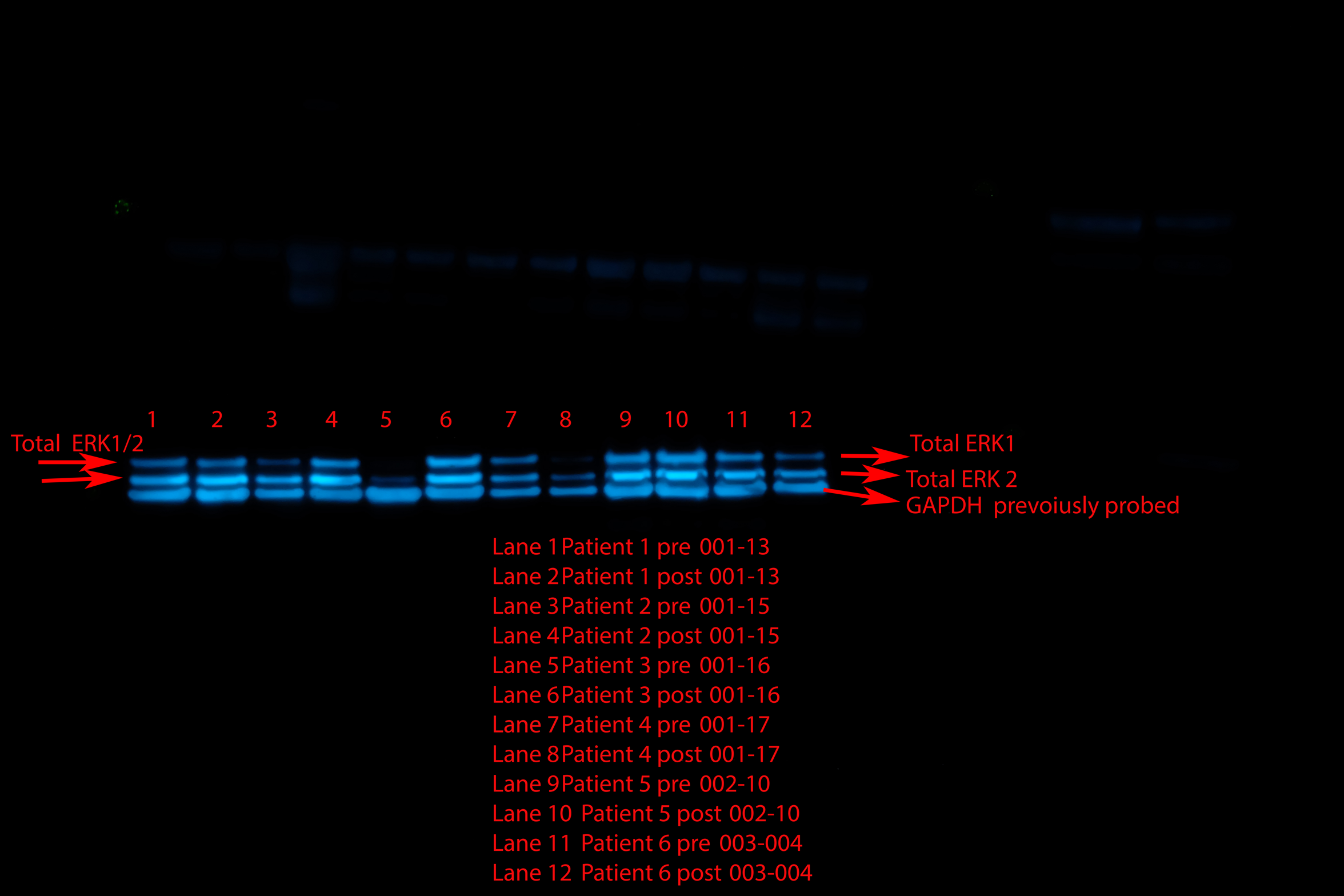

Supplement: Supplementary file 1 [file cancers-13-05504-s001.zip › orginal blots/Blots for 001-13, 001-15,001-16,001-17,002-10,003-004/TOTAL ERK12 PDF.pdf]
